# Efficacy and Safety of Hydroxychloroquine for Hospitalized COVID-19 Patients: A Systematic Review and Meta-Analysis

**DOI:** 10.3390/jcm10112503

**Published:** 2021-06-05

**Authors:** Adrian V. Hernandez, Mi T. Phan, Jonathon Rocco, Vinay Pasupuleti, Joshuan J. Barboza, Alejandro Piscoya, Yuani M. Roman, Charles M. White

**Affiliations:** 1 Health Outcomes, Policy and Evidence Synthesis (HOPES) Group, School of Pharmacy, University of Connecticut, Storrs, CT 06269, USA; mi.phan@uconn.edu (M.T.P.); jonathon.rocco@uconn.edu (J.R.); yuanniroman@gmail.com (Y.M.R.); charles.white@uconn.edu (C.M.W.); 2 Unidad de Revisiones Sistemáticas y Meta-análisis (URSIGET), Vicerrectorado de Investigación, Universidad San Ignacio de Loyola (USIL), Lima 15024, Peru; jbarbozameca@relaped.com (J.J.B.); alepiscoya@gmail.com (A.P.); 3 Cello Health, Yardley, PA 19067, USA; lepiscean@gmail.com; 4 Department of Research Administration, Hartford Hospital, Hartford, CT 06102, USA

**Keywords:** hydroxychloroquine, COVID-19, mortality, hospitalization, mechanical ventilation

## Abstract

We systematically reviewed the efficacy and safety of hydroxychloroquine as treatment for hospitalized COVID-19. Randomized controlled trials (RCTs) evaluating hydroxychloroquine as treatment for hospitalized COVID-19 patients were searched until 2nd of December 2020. Primary outcomes were all-cause mortality, need of mechanical ventilation, need of non-invasive ventilation, ICU admission and oxygen support at 14 and 30 days. Secondary outcomes were clinical recovery and worsening, discharge, radiological progression of pneumonia, virologic clearance, serious adverse events (SAE) and adverse events. Inverse variance random effects meta-analyses were performed. Thirteen RCTs (*n*=18,540) were included. Hydroxychloroquine total doses ranged between 2000 and 12,400 mg; treatment durations were from 5 to 16 days and follow up times between 5 and 30 days. Compared to controls, hydroxychloroquine non-significantly increased mortality at 14 days (RR 1.07, 95%CI 0.92–1.25) or 30 days (RR 1.08, 95%CI 1.00–1.16). Hydroxychloroquine did not affect other primary or secondary outcomes, except SAEs that were significantly higher than the control (RR 1.24, 95%CI 1.05–1.46). Eleven RCTs had high or some concerns of bias. Subgroup analyses were consistent with main analyses. Hydroxychloroquine was not efficacious for treating hospitalized COVID-19 patients and caused more severe adverse events. Hydroxychloroquine should not be recommended as treatment for hospitalized COVID-19 patients.

## 1. Introduction

As of the 22nd of February 2021, over 110 million people have been diagnosed with COVID-19, resulting in the death of ~2.5 million individuals [1]. Treatment options for COVID-19 are currently limited, with few treatment recommendations from the National Institutes of Health (NIH). The current guidance includes the use of corticosteroids in hospitalized patients requiring supplemental oxygen or remdesivir in hospitalized patients requiring non-mechanical oxygen supplementation [2].

Hydroxychloroquine (HCQ), an oral drug used for severe forms of rheumatoid arthritis and lupus erythematosus, has in vitro antiviral efficacy against coronaviruses including SARS-CoV-2 and immune modulating effects [3,4,5,6,7] and was among the first COVID-19 treatments investigated in human studies. HCQ, however, has several known adverse events such as QTc prolongation, cardiomyopathy, hypoglycemia and retinal toxicity in the non-COVID-19 environment [8]. By late Spring 2020, most of the evidence assessing HCQ efficacy and safety in hospitalized patients were from observational studies and showed widely divergent findings [9]. Since that time, several randomized controlled trials (RCTs) have been completed and provided a much clearer picture of the role of hydroxychloroquine in the treatment of hospitalized patients with COVID-19.

We systematically assessed RCTs evaluating HCQ effects vs. controls on clinical and safety outcomes in hospitalized COVID-19 patients. We also assessed quality of evidence of all outcomes using GRADE methodology.

## 2. Materials and Methods

### 2.1. Data Sources and Searches

Two investigators (V.P. and A.V.H.) developed the search strategy, which was revised and approved by the other investigators. We searched the following databases until 2nd of December 2020: PubMed-MEDLINE, EMBASE-OVID, Scopus, Web of Science, the Cochrane Library, medRxiv.org (www.medrxiv.org, accessed on 2 December 2020) and Preprints (www.preprints.org, accessed on 2 December 2020). The PubMed search strategy is shown in the Supplementary Materials.

### 2.2. Study Selection

Randomized controlled trials (RCTs) in any language reporting benefit or harm outcomes from use of hydroxychloroquine vs. control (placebo or usual care) as treatment in hospitalized with reverse transcription-polymerase chain reaction (RT-PCR)-confirmed COVID-19. We excluded studies in COVID-19 outpatients, studies of prophylaxis with hydroxychloroquine (i.e., in those without COVID-19 disease) and studies evaluating chloroquine. Three investigators (A.V.H., V.P., Y.M.R.) independently screened each record title and abstract for potential inclusion. Three investigators (V.P., J.J.B., Y.M.R.) then read the full text of the records whose abstracts had been selected by at least one investigator. Discrepancies were resolved through discussion or by a fourth investigator (A.V.H.).

### 2.3. Outcomes

Primary outcomes were all-cause mortality, need of mechanical ventilation, need of non-invasive ventilation, ICU admission and oxygen support at 14 and 28 or 30 days. Secondary outcomes were clinical recovery and worsening, discharge, radiological progression of pneumonia, virologic clearance, serious adverse events (SAE) and adverse events. Clinical recovery and worsening were extracted as defined by authors.

### 2.4. Data Extraction

Two investigators (A.P., J.J.B.) independently extracted the following variables from studies: study setting, country, COVID-19 diagnosis, hydroxychloroquine dose and duration, type of control and description, length of stay, primary and secondary outcomes and time of follow up. Discrepancies were resolved through discussion or by a third investigator (A.V.H.).

### 2.5. Risk of Bias Assessment

Three investigators (A.P., V.P., J.J.B.) independently assessed RoB by using the Cochrane Risk of Bias 2.0 tool for RCTs [10]; disagreements were resolved by discussion with a fourth investigator (A.V.H.). RoB per domain and study as low, some concerns and high for RCTs.

### 2.6. Statistical Analysis

We reported our systematic review according to 2009 PRISMA guidelines [11]. Inverse variance random effect meta-analyses were performed to evaluate effect of HCQ vs. control or SoC on outcomes when outcome data was available for at least two RCTs judged to have homogeneous study characteristics. Effects of meta-analyses were reported as relative risks (RR) for dichotomous outcomes; we also calculated their 95% confidence intervals (CIs). CIs of effects were adjusted with the Hartung-Knapp method [12], and the between study variance tau^2^ was calculated with the Paule-Mandel method [13]. Heterogeneity of effects among studies was quantified with the I^2^ statistic (an I^2^ > 60% means high heterogeneity). We pre-specified subgroup analyses by type of control and RoB; the p for interaction test <0.05 indicated effect modification by subgroup. The meta package of R 3.5.1 (www.r-project.org, accessed on 10 December 2020) was used for meta-analyses. The quality of evidence was evaluated using the GRADE methodology, which covers 5 aspects: risk of bias, inconsistency, indirectness, imprecision and publication bias [14]. Quality of evidence was evaluated per outcome and described in summary of findings (SoF) tables; GRADEpro GDT was used to create SoF tables [15].

## 3. Results

### 3.1. Selection of Studies

**Figure 1:**
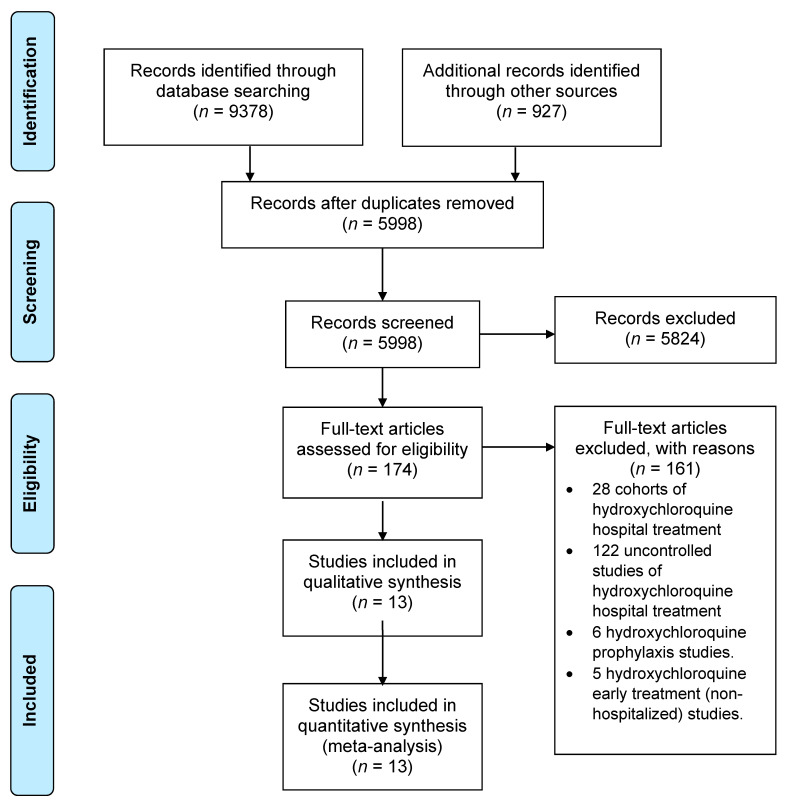
Flow chart of study selection.

Our comprehensive search yielded 9378 citations with an additional 927 citations identified through other sources, including backwards citation tracking. After removing duplicates and applying our inclusion and exclusion criteria (Figure 1), we identified thirteen RCTs (*n* = 18,540) [16,17,18,19,20,21,22,23,24,25,26,27,28].

### 3.2. Characteristics of Included Studies

The general characteristics of the included RCTs are included in Table 1. In most RCTs, all participants had COVID-19 confirmation via RT-PCR. However, patients in Chen, L. et al. [19], Cavalcanti et al. [20] and Horby et al. [21] had baseline RT-PCR positivity rates of 63%, 76% and 90%, respectively, and Chen, J. et al. [16] did not report the percentage of patients with RT-PCR positivity. All RCTs included adult populations. Placebo was the comparator in three RCTs (Self et al. [24], Dubee et al. [25], Ulrich et al. [28]) while usual care was the comparator in the other RCTs. The total dose of hydroxychloroquine in the RCTs varied between 2000 and 12,400 mg with follow up times between 5 and 30 days.

### 3.3. Risk of Bias of Included RCTs

**Figure 2:**
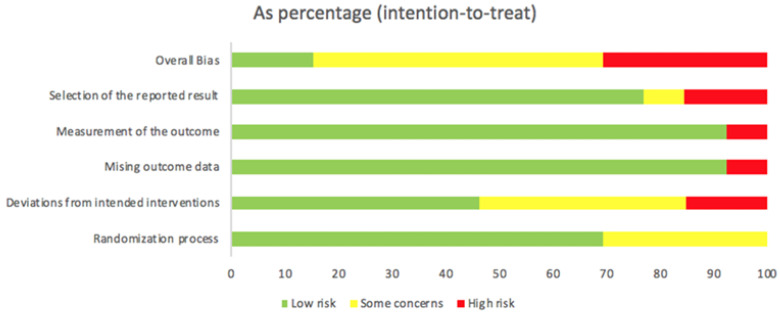
Risk of bias of included randomized controlled trials.

Four RCTs [18,19,20,23] had high risk of bias, one due to deviations from intended interventions, [18] one due to deviations from intended interventions and missing outcome data, [19] one due to measurement of the outcome, [20] and one due to selection of reported results [23]. Seven RCTs [16,17,22,24,26,27,28] had some concerns of bias, three due to the randomization process, [16,24,28] one due to randomization and deviation from intended interventions [26], two due to deviations from intended target, [22,27] and one due to selection of the reported results. [17] Two RCTs had low risk of bias in all categories [21,25] (Figure 2).

### 3.4. Effects of Hydroxichloroquine on Outcomes

**Figure 3:**
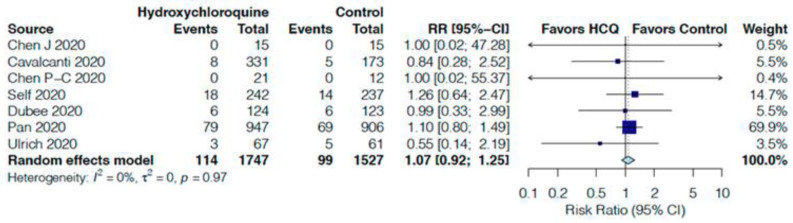
Effect of hydroxychloroquine on all-cause mortality at 14 days in hospitalized COVID-19 patients.

**Figure 4:**
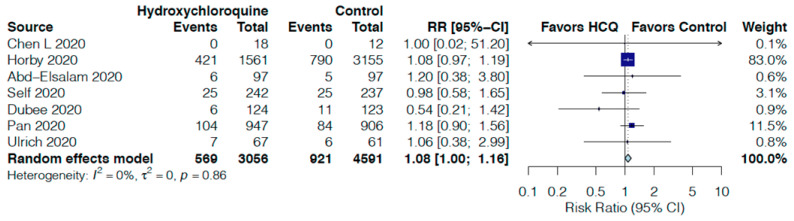
Effect of hydroxychloroquine on all-cause mortality at 30 days in hospitalized COVID-19 patients.

In comparison to the control group, HCQ non-significantly increased all-cause mortality by 7% (RR 1.07, 95%CI 0.92–1.25, I^2^ = 0%, Figure 3) at day 14 in seven RCTs [16,20,22,24,25,27,28] and by 8% (RR 1.08, 95%CI 1.00–1.16, I^2^ = 0%, Figure 4) at day 30 in seven RCTs [19,21,23,24,25,27,28]. HCQ did not impact the need for mechanical ventilation at 14 or 30 days (Supplementary Figures S1 and S2), high-flow nasal cannula or non-invasive ventilation at 14 days (Supplementary Figure S3), need for ICU admission (Supplementary Figure S4) and need of oxygen support at 14 days (Supplementary Figure S5) in comparison to the control group.

HCQ non-significantly increased the composite endpoint of clinical recovery by 26% in two RCTs [23,25] but with high heterogeneity (RR 1.26, 95%CI 0.87–1.83, I^2^ = 76%, Supplementary Figure S6) while HCQ also significantly increased the composite endpoint of clinical worsening by 14% in two RCTs [21,25] with no noted heterogeneity (RR 1.14, 95%CI 1.11–1.18, I^2^ = 0%, Supplementary Figure S7).

**Figure 5:**
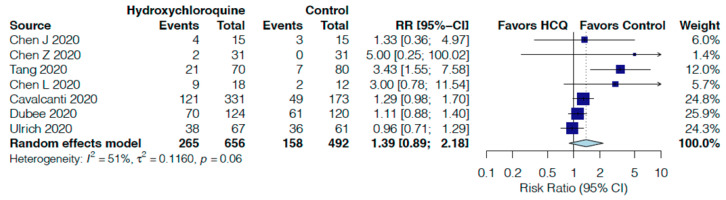
Effect of hydroxychloroquine on adverse events in hospitalized COVID-19 patients.

**Figure 6:**
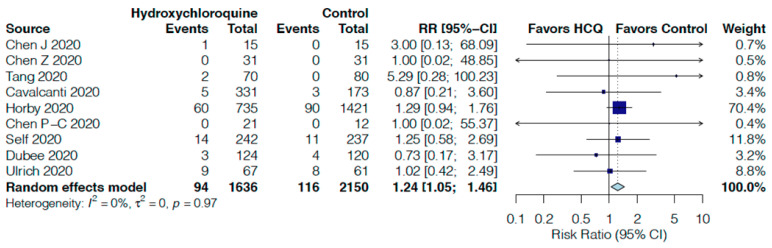
Effect of hydroxychloroquine on serious adverse events (SAEs) in hospitalized COVID-19 patients.

HCQ non-significantly increased the risk of overall adverse events by 39% (RR 1.39, 95%CI 0.89–2.18, I^2^ = 51%, Figure 5) in seven RCTs [16,17,18,19,20,25,28] and significantly increased serious adverse events by 24% (RR 1.24, 95%CI 1.05–1.46, I^2^ = 0%, Figure 6) in nine RCTs [16,17,18,20,21,22,24,25,28]. When each individual adverse event is assessed, no significant increases were found and HCQ did not impact radiological progression of pneumonia or virological clearance versus control (Supplementary Figures S9–S19).

### 3.5. Subgroup Analyses

Most of subgroup analyses by type of control (placebo or usual care) and RoB were consistent with main analyses (Supplementary Figure S20). No p for interaction tests were significant for clinical outcomes.

### 3.6. Quality of Evidence

The quality of evidence using the GRADE tool was high for HCQ causing clinical worsening; moderate for HCQ causing all-cause mortality at 14 and 30 days, need for mechanical ventilation at 30 days and serious adverse events; low for HCQ impacting need for supplementary oxygen and discharge from hospital; and very low for HCQ impacting need for mechanical ventilation at 14 days, need for high-flow nasal cannula or non-invasive ventilation, need for ICU admission, clinical recovery and overall adverse events (Table 2). Main drivers of low quality of evidence were individual studies with high or some concerns of bias, imprecision of effects and inconsistency of effects between RCTs.

## 4. Discussion

Since the time of our last systematic review update on HCQ in hospitalized patients, [9,29] the literature set is now robust enough to warrant meta-analysis. The dataset now includes 13 RCTs with only 30% deemed to have a high risk of bias. We can now say with moderate confidence that there was a trend towards HCQ use increasing all-cause mortality by 7–8%, with high confidence that patients treated with HCQ were 14% more likely to experience clinical worsening over their hospital course, and with moderate confidence that patients receiving HCQ were 24% more likely to experience serious adverse events. We also can conclude with low confidence that there was a trend that HCQ use can increase the need for oxygen support by 26%, and with very low confidence HCQ use had a trend towards increasing overall adverse events by 39%, ICU admission by 36%, noninvasive nasal cannula or non-invasive ventilation by 6% and mechanical ventilation at 14 days by 98%. Of all the aforementioned endpoints, no statistical heterogeneity was found, except for overall adverse events and mechanical ventilation at 14 days where it was high in both cases.

With moderate confidence, HCQ showed a trend towards reducing the need for mechanical ventilation at 30 days by 7%, with low confidence HCQ showed a trend towards speeding hospital discharge by 3%, and with very low confidence HCQ showed a trend towards improving clinical recovery by 26%. Unfortunately, HCQ did not reduce the need for mechanical ventilation at 14 days, the hospital discharge analysis had moderate statistical heterogeneity, and the clinical recovery analysis had very high statistical heterogeneity.

Taken together, HCQ should not be used in hospitalized patients with COVID-19 because the RCTs completed to date did not demonstrate a favorable balance of benefits to harm. We found no significant benefits with HCQ therapy and patients were significantly more likely to clinically worsen and have serious adverse events when given HCQ. The trend towards HCQ increasing overall mortality is especially troubling. It is unlikely that future studies will reveal positive benefits for hydroxychloroquine that outpace the potential for harms. This is strongly supported by the results of our subgroup analyses where only analyzing RCTs with a true placebo group or selectively analyzing RCTs with lower risk of bias were not different than what we found in our full dataset analyses.

Unlike previous systematic reviews, [30,31,32] including our previous review [9] and final update, [29] we limited this systematic review with meta-analyses to RCTs because this study type is inherently stronger and less prone to biases. Cohort studies assessing HCQ in COVID-19 have extensive clinical and methodological heterogeneity, especially those conducted earlier in the pandemic where the projects were rushed, and publications of lower quality projects were more likely. Likewise, the results of cohort studies were heterogenous and hard to interpret. In instances where the strength of evidence for an outcome was very low, if the direction of effect for an outcome was the same in RCT and cohort study analyses, they bolstered each other. However, it was impossible in most cases to reconcile areas where the different study types showed directions of effect moving in different directions. In those cases, the hierarchy of evidence would still suggest that the data from RCTs would be more likely to approximate the actual effects.

Among the 13 RCTs we included in our systematic review, only Cavalcanti et al., RCT [20] assessed HCQ alone or HCQ + azithromycin (AZ) vs. control. For our primary analysis, we combined both HCQ arms. The systematic review by Fiolet et al. [32] assessed mortality effects of HCQ ± AZ vs. control. Authors combined three RCTs for the comparison HCQ vs. control: Cavalcanti et al. [20] (HCQ vs. usual care in hospitalized patients), Horby et al., RECOVERY [21] (HCQ vs. usual care in hospitalized patients), and Skipper et al., (HCQ vs. placebo in non-hospitalized patients). For the comparison HCQ + AZ vs. control, Fiolet et al., only assessed Cavalcanti et al. [20]. For both comparisons, there was no effect of HCQ ± AZ vs. control on mortality. Bayesian secondary analysis gave similar results to primary analyses [32].

A recent systematic review of mortality outcomes by Axfors et al. [33] evaluated ongoing, completed or discontinued RCTs on HCQ or chloroquine treatment for any COVID-19 patients until October 16, 2020. This inclusion criteria were therefore broader than our study. Axfors et al., evaluated HCQ in 26 RCTs (*n* = 10,012) and we evaluated 7 RCTs at 30 days (*n* = 7647); both analyses were dominated by the Horby et al., RECOVERY [21] and Pan et al., WHO SOLIDARITY [27] RCTs, which employed relatively high doses and included 4716 and 1853 patients, respectively. Effects of HCQ on all-cause mortality between Axfors et al., and our study were similar and pointed to increased risk vs. control: OR 1.11 (95% CI 1.02, 1.20) and RR at 30 days 1.08 (95%CI 1.00, 1.16), respectively, with little heterogeneity.

We looked at a wider variety of outcomes than other systematic reviews to ensure that there were not unique benefits or harms that might not be identified in narrower assessments. We felt this was vital in truly understanding the balance of benefits to harms and as a result of our systematic review and meta-analysis saying the balance for HCQ use is unfavorable. A final advantage of our new systematic is that the literature search is updated to 2 December 2020 and included all major RCTs published on efficacy of hydroxychloroquine in hospitalized patients.

Chloroquine, an antimalarial drug, was proposed as therapeutic agent for COVID-19 because they were observed to inhibit SARS-CoV-2 viral replication in vitro in primate cells [5]; however, a later study found that chloroquine did not inhibit infection of human lung cells with SARS-CoV-2 [34]. HCQ, a less toxic derivative of chloroquine, inhibits trained immunity in vitro in peripheral blood mononuclear cells, which may not be beneficial for the antiviral innate immune response to SARS-CoV-2 infection in patients [35]. Chloroquine also has been investigated in vitro as potential treatment for other viruses such as human immunodeficiency virus [36], human coronavirus OC43, enterovirus EV-A71, Zika virus, influenza A H5N1, hepatitis C virus and chikungunya virus [37]; however, these studies did not show beneficial effects.

Our study had several limitations. First, most of outcomes had low or very low quality of evidence mainly driven by high or some concerns of bias, imprecision of effects and heterogeneity of effects. Second, there was heterogeneity of definitions of clinical worsening and clinical improving among RCTs; this situation was particularly prevalent in older studies. Third, we did not assess individual adverse events or serious adverse events due to scarcity of reporting across RCTs. Finally, we did not evaluate the effect of adding azithromycin to hydroxychloroquine as there was only one RCT [20] evaluating such combination.

## 5. Conclusions

Hydroxychloroquine was not efficacious for treating hospitalized COVID-19 patients and caused more severe adverse events. Hydroxychloroquine should not be recommended as treatment for hospitalized COVID-19 patients.

## Figures and Tables

**Table 1:** Baseline characteristics of included randomized controlled trials.

Author, Year [Ref]/ Registration	Objective	Sample, Country, Population	Overall Key Patient Characteristics	Intervention	Comparison	Outcomes	Follow Up Time
Chen, J. et al., 2020 [16], NCT04261517	To provide a database for exploring the next step in the effectiveness and safety of HCQ sulfate for COVID-19.	30 (I: 15, C: 15), China, Confirmed COVID-19 patients who were hospitalized in Shanghai Public Health Clinical Center from 6 to 25 February 2020; Inclusion criteria: Age ≥18 years, COVID-19 was diagnosed according to the “diagnosis and treatment plan”.RT-PCR positive for COVID-19: NR%	Mean (SD) age: 49 (4) years Males: 70% Median (IQR) days from symptom onset to enrolment: NR No coexisting disease: 63%	Conventional treatment plus oral HCQ sulfate 400 mg qd for 5 days (total dose: 2000 mg).	Only conventional treatment, including bed rest, oxygen inhalation and symptomatic supportive treatment.	Primary: Virologic clearance of a throat swab, sputum or lower respiratory tract secretion on day 7 or death of the patient within 2 weeks. Secondary: Serious adverse drug event or a change in the subject’s condition within 2 weeks (heavy and critical).	14 days
Chen, Z. et al., 2020 [17], ChiCTR2000029559	To evaluate the efficacy of HCQ in the treatment of patients with COVID-19.	62 (I: 31, C: 31), China, Patients with confirmed COVID-19 (diagnosis and classification of COVID-19 based on the criteria of the China National Health Commission). Inclusion criteria: Age ≥18 years; RT-PCR positive for SARS-CoV-2; Chest CT with pneumonia; SaO2/SpO2 ratio >93% or PaO2/FiO2 ratio >300 mmHg under the condition in the hospital room (mild illness).RT-PCR positive for COVID-19: 100%	Mean (SD) age: 45 (15) years Males: 47% Median (IQR) days from symptom onset to enrolment: NR No coexisting disease: NR	Oral HCQ sulfate tablets (Shanghai Pharma) 400 mg/d (200 mg bid) between days 1 and 5 plus standard treatment (total dose: 2000 mg).	Standard treatment only (O_2_ therapy, antiviral agents, antibacterial agents and immunoglobulin, with or without corticosteroids).	Primary: TTCR, changes in clinical characteristics. Secondary: radiological changes (chest CT) from day 0 to day 6.	5 days
Tang et al., 2020 [18], ChiCTR2000029868	To assess the efficacy and safety of HCQ plus standard-of-care compared with standard-of-care alone in adult patients with COVID-19.	150 (I: 75, C: 75), China, Patients (age ≥18 years) with ongoing SARS-CoV-2 infection confirmed in upper or lower respiratory tract specimens with RT-PCR and hospitalized in 16 hospitals in China. RT-PCR positive for COVID-19: 100%	Mean (SD) age: 46 (15) years Males: 55% Mean (SD) days from disease onset to randomization: 17 (11) No coexisting disease: 70%	Oral HCQ in a loading dose of 1200 mg qd for 3 days followed by a maintenance dose of 800 mg qd for remaining days (total duration: 2 weeks mild/moderate (99% patients) plus standard of care (total dose: 12,400 mg).	SOC (aligned with updated national guidelines for COVID-19 in China). No specific details of standard of care.	Primary: negative conversion of SARS-CoV-2 in respiratory specimens by 28 days; clinical improvement in severe COVID-19 by 28 days. Secondary: negative conversion of SARS-CoV-2 at 4, 7, 10, 14 and 21days; adverse events; alleviation of clinical symptoms, laboratory parameters, all-cause death; and chest radiology (all by 28days).	28 days
Chen, L. et al., 2020 [19], ChiCTR2000030054	To evaluate the clinical utility of CQ and HCQ in treating COVID-19.	67 (HCQ: 18, C: 12, CQ: 18, Excluded: 19), China, Individuals 18–75 years of age of Wuhan area, with mild or moderate COVID-19 based on the Chinese Diagnosis and Treatment Protocol for Novel Coronavirus Pneumonia (5th–7th Editions). Patients had positive RT-PCR test for SARS-CoV-2 or lung changes characteristic of COVID-19 on chest CT scan; at admission, all patients had SaO2 >93% at FiO2 21%. RT-PCR positive for COVID-19: 63%	Mean (SD) age: 48 (15) years Males: 47% Mean (SD) days from disease onset to randomization: NR No coexisting disease: 57%	Standard treatment plus oral HCQ sulfate at 200 mg bid for 10 days (total dose: 4000 mg).	Standard treatment only (No details provided).	Primary: TTCR. Secondary: Time to SARS-CoV-2 RNA negativity; length of hospital stay; changes on chest CT scan; days of supplemental oxygenation; adverse events; clinical status; all-cause mortality; vital signs; laboratory testing.	28 days
Cavalcanti et al., 2020 [20], NCT04322123	To assess whether HCQ, ± AZ, would be effective in improving clinical status at 15 days after hospital admission due to mild-to-moderate COVID-19.	665 (HCQ: 221, HCQ + AZ: 217, C: 227), Brazil, Patients ≥18 years of age who had been hospitalized with suspected or confirmed Covid-19 with 14 or fewer days since symptom onset.RT-PCR positive for COVID-19: 504 (75.8%) of all randomized (HCQ: 159, HCQ + AZ: 172, C: 173)	Mean (SD) age: 50 (15) years. Males: 58%. Median (IQR) days from symptom onset to randomization: 7 (5–9) No coexisting disease: 10%; O_2_ supplementation at baseline: 42%	SOC plus HCQ at 400 mg bid for 7 days (total dose: 5600 mg), or standard care plus HCQ at 400 mg bid plus AZ at 500 mg qd for 7 days (total dose: HCQ 5600 mg and AZ 3500 mg).	SOC (at the discretion of the treating physicians). Glucocorticoids, other immunomodulators, antibiotic agents and antiviral agents were allowed.	Primary: Clinical status using 7-point ordinal scale at 15 days. Secondary: Clinical status using 6-point ordinal scale; an indication for intubation; the receipt of supplemental oxygen administered; duration of hospital stay; in-hospital death; thromboembolic complications; acute kidney injury; the number of days alive and free from respiratory support.	15 days
Horby et al., RECOVERY, 2020 [21], NCT04381936	To evaluate the effects of HCQ versus usual care at 28 days in patients hospitalized with COVID-19.	4716 (HCQ: 1561, C: 3155), UK, Hospitalized patients with clinically suspected or laboratory confirmed SARS-CoV-2 infection and no medical history that might put patients at substantial risk if they were to participate in the trials. There was an age limit (i.e., only adults), but this was removed on May 9, 2020.RT-PCR positive for COVID-19: 90%	Mean (SD) age: 65 (15) years Males: 62% Median (IQR) days since symptom onset to randomization: 9 (5–14) No coexisting disease: 43%	Usual SOC plus HCQ sulfate tablets at 200 mg (containing 155-mg base equivalent) in a loading dose of 4 tablets at baseline and at 6 h, and followed by 2 tablets starting at 12 h after the initial dose and then bid for the next 9 days or until discharge, whichever occurred earlier (total dose: 9200 mg)	Usual SOC (unspecified).	Primary: All-cause mortality at 28 days. Secondary: Time until discharge from the hospital, a composite of the initiation of invasive MV (extracorporeal membrane oxygenation, death among patients who were not receiving invasive MV at the time of randomization).	28 days
Chen, C.-P. et al., 2020 [22], No registration	To evaluate HCQ efficacy and tolerability in adult patients with mild to moderate COVID-19.	33 (HCQ: 21, SOC: 12), Taiwan, Patients positive for SARS-CoV-2 infection RT-PCR of 11 public hospitals in northern, central and southern Taiwan affiliated with the Ministry of Health and Welfare. RT-PCR positive for COVID-19: 100%	Mean (SD) age: 33 (11) years Males: 63% Mean (SD) days from disease onset to randomization: NR No coexisting disease: NR	SOC plus HCQ 400 mg bid on day 1 and 200 mg BID for 6 days (total dose: 3200 mg).	SOC: (1) ceftriaxone 2 g qd for 7 days ± AZ 500 mg on day 1 and 250 mg on days 2–5; or (2) levofloxacin 750 mg qd for 5 d; or (3) levofloxacin 500 mg qd; or (4) moxifloxacin 400 mg qd for 7–14 days for subjects allergic to ceftriaxone or AZ.	Primary: Time to negative RT-PCR assessments at 14 days. Secondary: Proportion of negative viral RT-PCR on hospital day 14, resolution of clinical symptoms (time to clinical recovery), proportion of discharges by day 14, mortality rate.	14 days
Abd-Elsalam et al., 2020 [23], NCT04353336	To evaluate the safety and efficacy of HCQ added to the standard of care versus the standard of care alone in patients with COVID-19.	194 (HCQ + SOC: 97, SOC: 97), Egypt, Mild, moderate and severe (per WHO guidelines of March 2020) patients admitted to three tertiary centers with confirmed COVID-19 (i.e., SARS-CoV-2 infection) between March and June 2020.RT-PCR positive for COVID-19: 100%	Mean (SD) age: 40 (19) years Males: 59% Mean (SD) days from disease onset to randomization: NR No coexisting disease: 84%	SOC plus HCQ 400 mg BID in day 1 followed by 200 mg tablets bid for 15 days (total dose: 6800 mg).	SOC for 15d (paracetamol, O_2_ fluids), empiric antibiotic (cephalosporins), oseltamivir if needed (75 mg bid for 5 days), invasive MV with hydrocortisone for severe cases (PaO_2_ <60 mmHg, O_2_ saturation <90% despite O_2_ or NIV, progressive hypercapnia, respiratory acidosis progressive or refractory septic shock.	Primary: Recovery within 28 days, need for mechanical ventilation or death. Secondary: Duration to negative PCR, duration to clinical improvement, duration to hospital discharge.	28 days
Self et al., ORCHID, 2020 [24], NCT04332991	To test the hypothesis that, compared with placebo, HCQ improves clinical outcomes for adults hospitalized with COVID-19.	479 (HCQ: 242, Placebo: 237), USA, Adults (aged ≥18 years) who were hospitalized for less than 48 h with laboratory-confirmed SARS-CoV-2 infection and symptoms of respiratory illness for less than 10 days.RT-PCR positive for COVID-19: 100%	Mean (SD) age: 57 (18) years Males: 56% Median (IQR) days of symptoms to randomization: 5 (3–7) No coexisting disease: NR	400 mg HCQ sulfate pills bid for the first 2 doses followed by 200 mg bid for the subsequent 8 doses, for a total of 10 doses over 5 days (total dose: 2400 mg).	Matching placebo in the same dosing frequency.	Primary: Clinical status 14 days after randomization assessed with a 7-category ordinal scale (COVID Outcomes Scale) by the WHO. Secondary: Scores on the COVID Outcomes Scale, all-cause all-location mortality, time to recovery, composite of death or receipt of ECMO, support-free days.	28 days
Dubee et al., HYCOVID, 2020 [25], NCT04325893	To evaluate the efficacy and safety of HCQ in adult patients with mild-to-moderate COVID-19 at risk of worsening.	250 (HCQ: 125, Placebo: 125), France/Monaco, Patients with COVID-19 confirmed by positive SARS-CoV-2 RT-PCR or positive CT scan and had ≥1 of the following risk factors for worsening: (i) age ≥75 years; (ii) age 60–74 years and presence of ≥1 of the following comorbidities: obesity (BMI ≥30 kg/m²), arterial hypertension requiring treatment, diabetes mellitus requiring treatment; (iii) need for O2 to reach a peripheral capillary SpO2 >94% or a PaO2/FiO2 ≤300 mmHg.RT-PCR positive for COVID-19: 99%	Mean (SD) age: 75 (21) years Males: 48% Median (IQR) days from onset to inclusion: 5 (3–9) No coexisting disease: NR	SOC plus oral HCQ (200 mg tablets, orally) at a dose of 2 tablets bid for day 1 followed by 1 tablet bid for8 days (total dose: 4000 mg).	Matching placebo at a dose of 2 tablets bid in day 1 followed by 1 tablet bid for 8 days plus SOC as needed.	Primary: Composite of death, the need for invasive MV within 14 days after randomization. Secondary: Mortality and clinical evolution at day 14 and 28, viral shedding at day 5 and 10.	28 days
Kamran et al., 2020 [26], NCT04491994	To analyze the efficacy of HCQ in addition to standard of care compared with standard of care alone in reducing disease progression in mild COVID-19.	500 (HCQ + SOC: 349, SOC: 151), Pakistan, Hospitalized patients aged 18–80 years from both genders with Mild confirmed COVID-19 (positive RT-PCR of oropharyngeal and nasopharyngeal swabs). Mild disease was defined per WHO criteria.RT-PCR positive for COVID-19: 100%	Mean (SD) age: 34 (11) years Males: 93% Mean (SD) days from disease onset to randomization: NR No coexisting disease: NR	SOC plus oral HCQ 400 mg bid for day 1 followed by 200 mg bid for next 5 days (total dose: 2800 mg).	SOC (daily oral vitamin C, oral zinc, oral vitamin D, paracetamol and intravenous fluids).	Primary: Disease progression within 5 days of start of treatment (i.e., development of fever >101 F for >72 h, shortness of breath by minimal exertion (10- Step walk test), derangement of basic lab parameters (ALC <1000 or raised CRP) or appearance of infiltrates on CXR). Secondary: Viral clearance, PCR negativity on day 7 and 14 after admission was recorded.	14 days
Pan et al., SOLIDARITY, 2020 [27], NCT04315948	To help determine whether any of four repurposed antivirals could at least moderately affect in hospital mortality.	11266 (HCQ: 954, no drug (SOC):4088, other drugs: 6288), 30 countries worldwide, Patients 18 years of age or older, were hospitalized with a diagnosis of Covid-19, were not known to have received any trial drug, were not expected to be transferred elsewhere within 72 h and, in the physician’s view, had no contraindication to any trial drug.RT-PCR positive for COVID-19: 100%	Age <70 years: 79%. Mean (SD): NR Males: 62% Mean (SD) days from disease onset to randomization: NR No coexisting disease: 44%	Oral HCQ sulfate 200 mg tablets (containing 155-mg base equivalent) at a dose of 4 tablets at hour 0, 4 tablets at hour 6. Starting at hour 12, 2 tablets bid for 10 days (total dose: 9600 mg).	SOC. No details of standard of care. Other interventions (remdesivir, lopinavir, interferon).	Primary: In-hospital mortality (i.e., death during the original hospitalization; follow-up ceased at discharge), regardless of whether death occurred before or after day 28. Secondary: Initiation of MV, hospitalization duration.	28 days
Ulrich et al., TEACH, 2020 [28], NCT04369742	To evaluate the efficacy and safety of HCQ in hospitalized patients with COVID-19. We hypothesized that HCQ is superior to placebo in preventing severe outcomes among hospitalized COVID-19 patients.	128 (HCQ: 67, Placebo: 61), USA, Hospitalized patients with a positive RT-PCR for SARS-CoV-2 within 72 h of enrollment, and at least one COVID-19 symptom (e.g., fever, cough, dyspnea, nausea, diarrhea, myalgia, anosmia, dysgeusia) and the subject’s (or legally authorized representative’s), written informed consent.RT-PCR positive for COVID-19: 100%.	Mean (SD) age: 66 (16) years Males: 59% Median (IQR) days since symptom onset: 7 (10) No coexisting disease: 13%	Oral HCQ 400 mg (2 tablets) bid for day 1 and 200 mg (1 tablet) bid for days 2–5 (total dose: 2400 mg).	Placebo: calcium citrate 200-mg tablets, same schedule as HCQ.	Primary: Proportion of subjects meeting a severe COVID-19 progression composite end point (death, ICU admission, VM, ECMO and/or vasopressor use) at day 14. The primary safety outcome was serious adverse events (SAEs), grade 3 or 4 adverse events and/or discontinuation of therapy at day 30. Secondary: changes in an 8-point ordinal COVID-19 clinical severity score, the primary composite outcome and mortality at day 30, hospital LOS, fever-free days, oxygen-free days, laboratory outcomes.	30 days

HCQ: Hydroxychloroquine; CQ: Chloroquine; AZ: Azithromycin; I: Intervention group; C: Control group; SOC: Standard of care; RT-PCR: Reverse transcription-polymerase chain reaction; SARS-CoV-2: Severe acute respiratory syndrome coronavirus 2; SD: Standard deviation; IQR: Interquartile range; NR: Not reported; CT: Computed tomography; BMI: Body mass index; TTCR: Time to clinical recovery; ICU: Intensive care unit; MV: Mechanical ventilation; NIV: Non-invasive ventilation; ECMO: Extracorporeal membrane oxygenation; LOS: Length of stay; O_2_: Oxygen supplementation; ALC: Absolute lymphocyte count; CRP: C-reactive protein; CXR: Chest X rays.

**Table 2:** Summary of findings (SoF) table of the effects of hydroxychloroquine on outcomes of hospitalized COVID-19 patients.

Outcomes	Anticipated Absolute Effects ^*^ (95% CI)		Relative Effect (95% CI)	№ of Participants (Studies)	Certainty of the Evidence (GRADE)
	Risk with Control	Risk with Hydroxychloroquine			
All-cause mortality follow-up: mean 14 days	6 per 100	7 per 100	RR 1.07	3274	⨁⨁⨁◯
		(6 to 8)	(0.92 to 1.25)	(7 RCTs)	MODERATE ^a^
All-cause mortality follow-up: mean 30 days	20 per 100	22 per 100	RR 1.08	7647	⨁⨁⨁◯
		(20 to 23)	(1.00 to 1.16)	(7 RCTs)	MODERATE ^b^
Need for mechanical ventilation follow up: mean 14 days	2 per 100	3 per 100	RR 1.98	1419	⨁◯◯◯
		(0 to 25)	(0.24 to 16.22)	(3 RCTs)	VERY LOW ^c–e^
Need for mechanical ventilation follow up: mean 30 days	5 per 100	4 per 100	RR 0.93	1048	⨁⨁⨁◯
		(3 to 7)	(0.61 to 1.41)	(4 RCTs)	MODERATE ^f^
Need for high-flow nasal cannula or non-invasive ventilation follow up: mean 14 days	2 per 100	2 per 100	RR 1.06	1111	⨁◯◯◯
		(0 to 25)	(0.11 to 10.69)	(3 RCTs)	VERY LOW ^g–i^
Need for ICU admission follow up: range 14 days to 30 days	10 per 100	14 per 100	RR 1.36	322	⨁◯◯◯
		(4 to 42)	(0.44 to 4.19)	(2 RCTs)	VERY LOW ^j–l^
Need for supplementary oxygen follow up: mean 14 days	5 per 100	6 per 100	RR 1.26	1111	⨁⨁◯◯
		(3 to 14)	(0.58 to 2.73)	(3 RCTs)	LOW ^g,m^
Clinical recovery assessed with: Alleviation of clinical symptoms, three consecutive negative PCR tests, discharge from hospital alive OR better ordinal scale at follow up (5 to 7 in 7-point scale or 0 to 2 in 8-point scale) follow up: mean 30 days	53 per 100	67 per 100	RR 1.26	441	⨁◯◯◯
		(46 to 97)	(0.87 to 1.83)	(2 RCTs)	VERY LOW ^n,o^
Clinical worsening assessed with: Death or invasive mechanical ventilation follow up: mean 30 days	26 per 100	30 per 100	RR 1.14	4170	⨁⨁⨁⨁
		(29 to 31)	(1.11 to 1.18)	(2 RCTs)	HIGH
Discharge from hospital follow up: range 14 days to 30 days	64 per 100	62 per 100	RR 0.97	5569	⨁⨁◯◯
		(53 to 73)	(0.83 to 1.13)	(4 RCTs)	LOW ^p,q^
Adverse events follow up: range 5 days to 30 days	32 per 100	45 per 100	RR 1.39	1148	⨁◯◯◯
		(29 to 70)	(0.89 to 2.18)	(7 RCTs)	VERY LOW ^r,s^
Serious adverse events follow up: range 5 days to 30 days	5 per 100	7 per 100	RR 1.24	3786	⨁⨁⨁◯
		(6 to 8)	(1.05 to 1.46)	(9 RCTs)	MODERATE ^t^

^a^. RoB: Chen, J. et al., has some concerns of bias in the randomization process; Cavalcanti et al., has high risk of bias in the selection of the reported result; Chen, C.-P. et al., has some concerns in deviations from intended interventions; Self et al., has some concerns in the randomization process; Dubee et al., has low risk of bias; Pan et al., has some concerns in deviations from intended interventions; Ulrich et al., has some concerns in the randomization process. ^b^. RoB: Chen, L. et al., has high risk in missing outcome data; Horby et al., has low risk of bias; Abd-Elsalam et al., has high of bias in the measurement of the outcome; Self et al., has some concerns in the randomization process; Dubee et al., has low risk of bias; Pan et al., has some concerns in deviations from intended interventions; Ulrich et al., has some concerns in the randomization process. ^c^. RoB: Cavalcanti et a. has high risk of bias in the selection of the reported result; Dubee et al., has low risk of bias; Ulrich et al., has some concerns of bias in the randomization process. ^d^. Inconsistency: I2 = 52% ^e^. Imprecision: 95%CI of effects is 0.24 to 16.22 ^f^. RoB: Abd-Elsalam et al., has high of bias in the measurement of the outcome; Self et al., has some concerns in the randomization process; Dubee et al., has low risk of bias; Ulrich et al., has some concerns of bias in the randomization process. ^g^. RoB: Cavalcanti et a. has high risk of bias in the selection of the reported result; Self et al., has some concerns of bias in the randomization process; Ulrich et al., has some concerns of bias in the randomization process. ^h^. Inconsistency: I2 = 33% ^i^. Imprecision: 95%CI of effect is 0.11 to 10.69 ^j^. Rob: Abd-Elsalam et al., had high risk of bias in the measurement of the outcome; Ulrich et al., has some concerns of bias in the randomization process. ^k^. Inconsistency: I2 = 59% ^l^. Imprecision: 95%CI of effect is 0.44 to 4.19 ^m^. Imprecision: 95%CI of effect is 0.58 to 2.73 ^n^. RoB: Abd-Elsalam et al., had high risk of bias in the measurement of the outcome; Dubee et al., had low risk of bias. ^o^. Inconsistency: I2 = 76% ^p^. RoB: Horby et al., and Dubee et al., have low risk of bias; Self et al. and Ulrich et al., have some concerns of bias in the randomization process. ^q^. Inconsistency: I2 = 44% ^r^. RoB: Chen, J. et al., and Ulrich et al., have some concerns in randomization process; Chen, Z. et al., has some concerns in the selection of the reported result; Tang et al., has high risk in deviations from intended interventions and selection of the reported result; Chen, L. et al., has high risk in deviations from intended interventions and missing outcome data; Cavalcanti et al., has high risk in the selectio of the reported result; Dubee et al., has low risk of bias. ^s^. Inconsistency: I2 = 51%. ^t^. RoB: Chen, J. et al., Self et al. and Ulrich et al., have some concerns in randomization process; Chen Z et al., has some concerns in the selection of the reported result; Tang et al., has high risk in deviations from intended interventions and selection of the reported result; Chen, C.-P. et al., has some concerns in deviations from intended interventions; Cavalcanti et al., has high risk in the selection of the reported result; Horby et al., and Dubee et al., have low risk of bias.

## Supplementary Materials

The following are available online at https://www.mdpi.com/article/10.3390/jcm10112503/s1, Figure S1: Effect of hydroxychloroquine on need for mechanical ventilation at 14 days in hospitalized COVID-19 patients; Figure S2: Effect of hydroxychloroquine on need for mechanical ventilation at 30 days in hospitalized COVID-19 patients; Figure S3: Effect of hydroxychloroquine on need for high-flow nasal cannula or non-invasive ventilation at 14 days in hospitalized COVID-19 patients; Figure S4: Effect of hydroxychloroquine on need for ICU admission in hospitalized COVID-19 patients; Figure S5: Effect of hydroxychloroquine on need for oxygen support at 14 days in hospitalized COVID-19 patients; Figure S6: Effect of hydroxychloroquine on clinical recovery in hospitalized COVID-19 patients; Figure S7: Effect of hydroxychloroquine on clinical worsening (death or invasive mechanical ventilation) in hospitalized COVID-19 patients; Figure S8: Effect of hydroxychloroquine on discharge in hospitalized COVID-19 patients; Figure S9: Effect of hydroxychloroquine on radiological progression of pneumonia in hospitalized COVID-19 patients; Figure S10: Effect of hydroxychloroquine on virologic clearance at 14 days in hospitalized COVID-19 patients; Figure S11: Effect of hydroxychloroquine on gastrointestinal adverse events (nausea, vomiting, abdominal pain) in hospitalized COVID-19 patients; Figure S12: Effect of hydroxychloroquine on abnormal liver function in hospitalized COVID-19 patients; Figure S13: Effect of hydroxychloroquine on rash in hospitalized COVID-19 patients; Figure S14: Effect of hydroxychloroquine on headache in hospitalized COVID-19 patients; Figure S15: Effect of hydroxychloroquine on QTc prolongation in hospitalized COVID-19 patients; Figure S16: Effect of hydroxychloroquine on anemia in hospitalized COVID-19 patients; Figure S17: Effect of hydroxychloroquine on ventricular tachycardia in hospitalized COVID-19 patients; Figure S18: Effect of hydroxychloroquine on leukopenia in hospitalized COVID-19 patients; Figure S19: Effect of hydroxychloroquine on lymphocytopenia in hospitalized COVID-19 patients; Figure S20: Subgroup analyses by type of control and risk of bias for all outcomes in hospitalized COVID-19 patients.
