# Supplementary material for: Efficacy and Safety of Hydroxychloroquine for Hospitalized COVID-19 Patients: A Systematic Review and Meta-Analysis
Source: J Clin Med. 2021 Jun 5;10(11):2503. doi: 10.3390/jcm10112503 (PMC8201261; doi:10.3390/jcm10112503)
Supplement: Supplementary file 1 [file jcm-10-02503-s001.zip › jcm-1234977-supplementary.pdf]

Supplementary Materials
Pubmed search strategy

Supplementary Figure 1: Effect of hydroxychloroquine on need for mechanical ventilation at 14 days in hospitalized COVID-19 patients

Supplementary Figure 2: Effect of hydroxychloroquine on need for mechanical ventilation at 30 days in hospitalized COVID-19 patients

Supplementary Figure 3: Effect of hydroxychloroquine on need for high-flow nasal cannula or non-invasive ventilation at 14 days in hospitalized COVID-19 patients

Supplementary Figure 4: Effect of hydroxychloroquine on need for ICU admission in hospitalized COVID-19 patients

Supplementary Figure 5: Effect of hydroxychloroquine on need for oxygen support at 14 days in hospitalized COVID-19 patients

Supplementary Figure 6: Effect of hydroxychloroquine on clinical recovery in hospitalized COVID-19 patients

Supplementary Figure 7: Effect of hydroxychloroquine on clinical worsening (death or invasive mechanical ventilation) in hospitalized COVID-19 patients

Supplementary Figure 8: Effect of hydroxychloroquine on discharge in hospitalized COVID-19 patients

Supplementary Figure 9: Effect of hydroxychloroquine on radiological progression of pneumonia in hospitalized COVID-19 patients

Supplementary Figure 10: Effect of hydroxychloroquine on virologic clearance at 14 days in hospitalized COVID-19 patients

Supplementary Figure 11: Effect of hydroxychloroquine on gastrointestinal adverse events (nausea, vomiting, abdominal pain) in hospitalized COVID-19 patients

Supplementary Figure 12: Effect of hydroxychloroquine on abnormal liver function in hospitalized COVID-19 patients

Supplementary Figure 13: Effect of hydroxychloroquine on rash in hospitalized COVID-19 patients

Supplementary Figure 14: Effect of hydroxychloroquine on headache in hospitalized COVID-19 patients

Supplementary Figure 15: Effect of hydroxychloroquine on QTc prolongation in hospitalized COVID-19 patients

Supplementary Figure 16: Effect of hydroxychloroquine on anemia in hospitalized COVID-19 patients

Supplementary Figure 17: Effect of hydroxychloroquine on ventricular tachycardia in hospitalized COVID-19 patients

Supplementary Figure 18: Effect of hydroxychloroquine on leukopenia in hospitalized COVID-19 patients.

Supplementary Figure 19: Effect of hydroxychloroquine on lymphocytopenia in hospitalized COVID-19 patients.

Supplementary Figure 20: Subgroup analyses

Pubmed search strategy

("hydroxychloroquine"[MeSH Terms] OR "hydroxychloroquine"[All Fields]) AND ("covid 19"[All Fields] OR "covid 19"[MeSH Terms] OR "covid 19 vaccines"[All Fields] OR "covid 19 vaccines"[MeSH Terms] OR "covid 19 serotherapy"[All Fields] OR "covid 19 serotherapy"[Supplementary Concept] OR "covid 19 nucleic acid testing"[All Fields] OR "covid 19 nucleic acid testing"[MeSH Terms] OR "covid 19 serological testing"[All Fields] OR "covid 19 serological testing"[MeSH Terms] OR "covid 19 testing"[All Fields] OR "covid 19 testing"[MeSH Terms] OR "sars cov 2"[All Fields] OR "sars cov 2"[MeSH Terms] OR "severe

acute respiratory syndrome coronavirus 2"[All Fields] OR "ncov"[All Fields] OR "2019 ncov"[All Fields] OR (("coronavirus"[MeSH Terms] OR "coronavirus"[All Fields] OR "cov"[All Fields]) AND 2019/11/01:3000/12/31[Date - Publication])) AND ("randomized controlled trial"[Publication Type] OR "randomized controlled trials as topic"[MeSH Terms] OR "randomized controlled trial"[All Fields] OR "randomised controlled trial"[All Fields])

Supplementary Figure S1: Effect of hydroxychloroquine on need for mechanical ventilation at 14 days in hospitalized COVID-19 patients

	Hydroxychloroquine		Control				
**Source**	**Events**	**Total**	**Events**	**Total**	**RR [95%−CI]**	**Favors HCQ Favors Control**	**Weight**
Cavalcanti 2020	14	331	7	713	4.31 [1.76; 10.57]		43.9%
Dubee 2020	3	124	3	123	0.99 [0.20; 4.82]		24.3%
Ulrich 2020	5	67	4	61	1.14 [0.32; 4.05]		31.8%
**Random effects model**	**22**	**522**	**14**	**897**	**1.98 [0.24; 16.22]**		**100.0%**

Heterogeneity: $I^2 = 52\%$, $\tau^2 = 0.3353$, $p = 0.13$

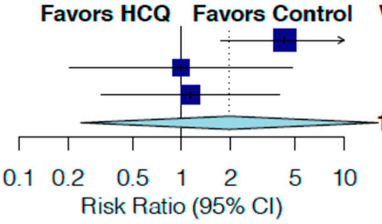

Supplementary Figure S2: Effect of hydroxychloroquine on need for mechanical ventilation at 30 days in hospitalized COVID-19 patients

	Hydroxychloroquine		Control				
**Source**	**Events**	**Total**	**Events**	**Total**	**RR [95%−CI]**	**Favors HCQ Favors Control**	**Weight**
Abd−Elsalam 2020	4	97	5	97	0.80 [0.22; 2.89]		19.2%
Self 2020	11	242	12	237	0.90 [0.40; 1.99]		49.8%
Dubee 2020	3	124	4	123	0.74 [0.17; 3.26]		14.6%
Ulrich 2020	5	67	3	61	1.52 [0.38; 6.08]		16.4%
**Random effects model**	**23**	**530**	**24**	**518**	**0.93 [0.61; 1.41]**		**100.0%**

Heterogeneity: $I^2 = 0\%$, $\tau^2 = 0$, $p = 0.89$

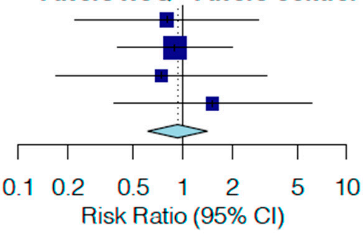

Supplementary Figure S3: Effect of hydroxychloroquine on need for high-flow nasal cannula or non-invasive ventilation at 14 days in hospitalized COVID-19 patients

	Hydroxychloroquine		Control				
**Source**	**Events**	**Total**	**Events**	**Total**	**RR [95%−CI]**	**Favors HCQ Favors Control**	**Weight**
Cavalcanti 2020	2	331	2	173	0.52 [0.07; 3.68]		22.4%
Self 2020	5	242	7	237	0.70 [0.23; 2.17]		45.8%
Ulrich 2020	7	67	2	61	3.19 [0.69; 14.76]		31.8%
**Random effects model**	**14**	**640**	**11**	**471**	**1.06 [0.11; 10.69]**		**100.0%**

Heterogeneity: $I^2 = 33\%$, $\tau^2 = 0.2948$, $p = 0.22$

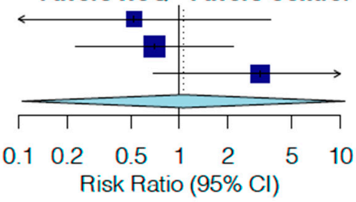

Supplementary Figure S4: Effect of hydroxychloroquine on need for ICU admission in hospitalized COVID-19 patients

	Hydroxychloroquine		Control			
**Source**	**Events**	**Total**	**Events**	**Total**	**RR [95%−CI]**	**Weight**
Abd–Elsalam 2020	11	97	13	97	0.85 [0.40; 1.79]	59.7%
Ulrich 2020	9	67	3	61	2.73 [0.77; 9.63]	40.3%
**Random effects model**	**20**	**164**	**16**	**158**	**1.36 [0.44; 4.19]**	**100.0%**

Heterogeneity: $I^2 = 59\%$, $\tau^2 = 0.4064$, $p = 0.12$

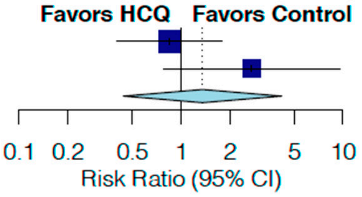

Supplementary Figure S5: Effect of hydroxychloroquine on need for oxygen support at 14 days in hospitalized COVID-19 patients

	Hydroxychloroquine		Control			
**Source**	**Events**	**Total**	**Events**	**Total**	**RR [95%−CI]**	**Weight**
Cavalcanti 2020	11	331	5	173	1.15 [0.41; 3.26]	23.4%
Self 2020	22	242	18	237	1.20 [0.66; 2.17]	71.2%
Ulrich 2020	4	67	1	61	3.64 [0.42; 31.69]	5.4%
**Random effects model**	**37**	**640**	**24**	**471**	**1.26 [0.58; 2.73]**	**100.0%**

Heterogeneity: $I^2 = 0\%$, $\tau^2 = 0$, $p = 0.61$

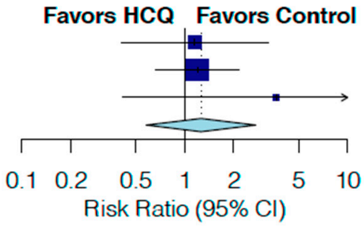

Supplementary Figure S6: Effect of hydroxychloroquine on clinical recovery in hospitalized COVID-19 patients

	Hydroxychloroquine		Control			
**Source**	**Events**	**Total**	**Events**	**Total**	**RR [95%−CI]**	**Weight**
Abd–Elsalam 2020	52	97	33	97	1.58 [1.13; 2.20]	42.4%
Dubee 2020	91	124	84	123	1.07 [0.92; 1.26]	57.6%
**Random effects model**	**143**	**221**	**117**	**220**	**1.26 [0.87; 1.83]**	**100.0%**

Heterogeneity: $I^2 = 76\%$, $\tau^2 = 0.0555$, $p = 0.04$

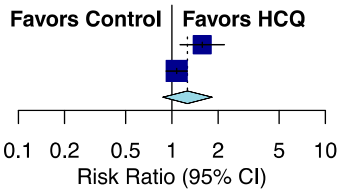

Supplementary Figure S7: Effect of hydroxychloroquine on clinical worsening (death or invasive mechanical ventilation) in hospitalized COVID-19 patients

Source	Hydroxychloroquine Events	Hydroxychloroquine Total	Control Events	Control Total	RR [95%–CI]	Weight
Horby 2020	399	1300	705	2623	1.14 [1.03; 1.27]	98.8%
Dubee 2020	9	124	8	123	1.12 [0.45; 2.80]	1.2%
**Random effects model**	**408**	**1424**	**713**	**2746**	**1.14 [1.11; 1.18]**	**100.0%**

Heterogeneity: $I^2 = 0\%$, $\tau^2 = 0$, $p = 0.96$

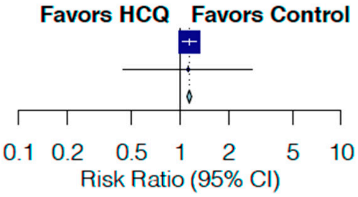

Supplementary Figure S8: Effect of hydroxychloroquine on discharge in hospitalized COVID-19 patients

Source	Hydroxychloroquine Events	Hydroxychloroquine Total	Control Events	Control Total	RR [95%–CI]	Weight
Horby 2020	931	1561	1983	3155	0.95 [0.90; 1.00]	38.9%
Self 2020	191	242	185	237	1.01 [0.92; 1.11]	30.3%
Dubee 2020	90	123	85	123	1.06 [0.90; 1.24]	19.2%
Ulrich 2020	41	67	47	61	0.79 [0.63; 1.00]	11.6%
**Random effects model**	**1253**	**1993**	**2300**	**3576**	**0.97 [0.83; 1.13]**	**100.0%**

Heterogeneity: $I^2 = 44\%$, $\tau^2 = 0.0052$, $p = 0.15$

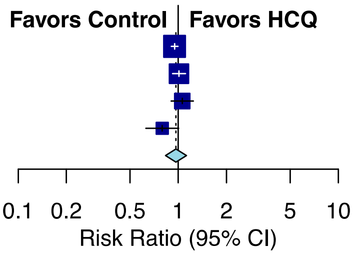

Supplementary Figure S9: Effect of hydroxychloroquine on radiological progression of pneumonia in hospitalized COVID-19 patients

Source	Hydroxychloroquine Events	Hydroxychloroquine Total	Control Events	Control Total	RR [95%–CI]	Weight
Chen J 2020	5	15	7	15	0.71 [0.29; 1.75]	33.2%
Chen Z 2020	25	31	17	31	1.47 [1.02; 2.11]	66.8%
**Random effects model**	**30**	**46**	**24**	**46**	**1.16 [0.59; 2.25]**	**100.0%**

Heterogeneity: $I^2 = 53\%$, $\tau^2 = 0.1388$, $p = 0.14$

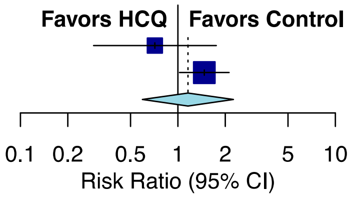

Supplementary Figure S10: Effect of hydroxychloroquine on virologic clearance at 14 days in hospitalized COVID-19 patients

Source	Hydroxychloroquine Events	Hydroxychloroquine Total	Control Events	Control Total	RR [95%–CI]	Weight
Chen J 2020	15	15	15	15	1.00 [0.88; 1.14]	43.6%
Chen P–C 2020	17	21	9	12	1.08 [0.73; 1.59]	4.8%
Kamran 2020	244	349	110	151	0.96 [0.85; 1.08]	50.6%
Ulrich 2020	8	67	10	61	0.73 [0.31; 1.73]	1.0%
**Random effects model**	**284**	**452**	**144**	**239**	**0.98 [0.91; 1.06]**	**100.0%**

Heterogeneity: $I^2 = 0\%$, $\tau^2 = 0$, $p = 0.82$

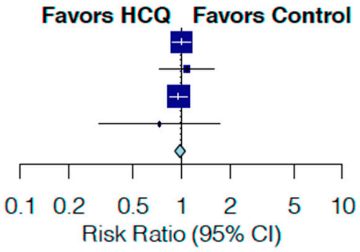

Supplementary Figure S11: Effect of hydroxychloroquine on gastrointestinal adverse events (nausea, vomiting, abdominal pain) in hospitalized COVID-19 patients

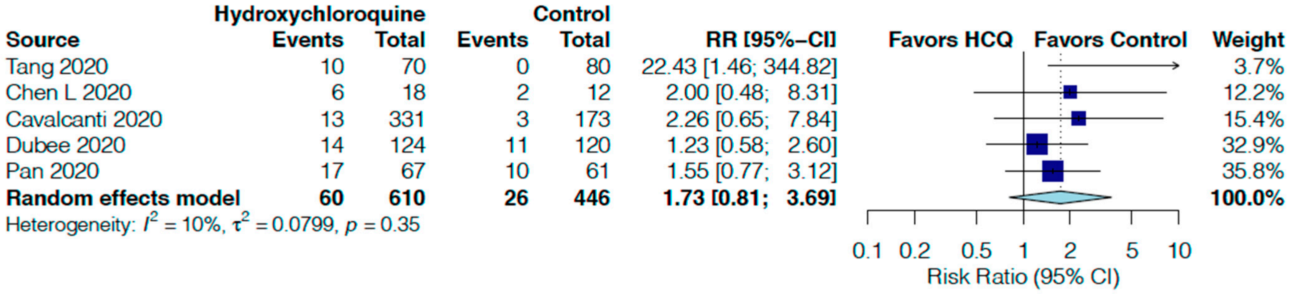

Supplementary Figure S12: Effect of hydroxychloroquine on abnormal liver function in hospitalized COVID-19 patients

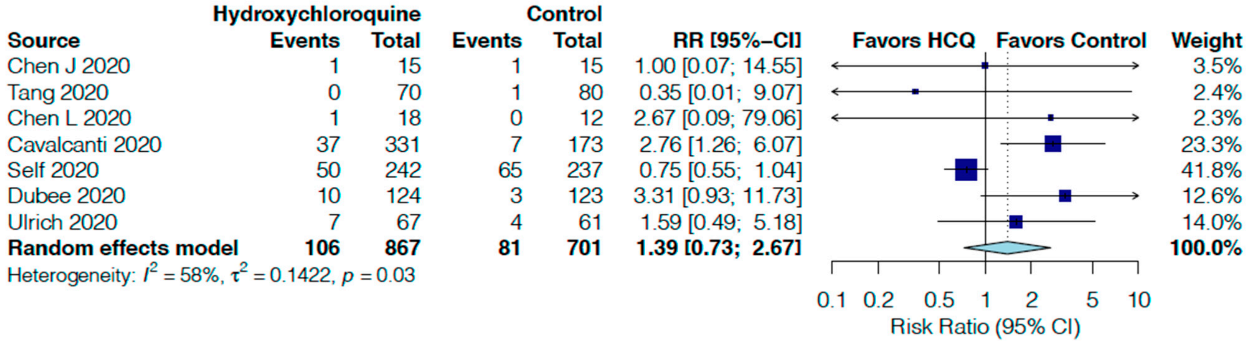

Supplementary Figure S13: Effect of hydroxychloroquine on rash in hospitalized COVID-19 patients

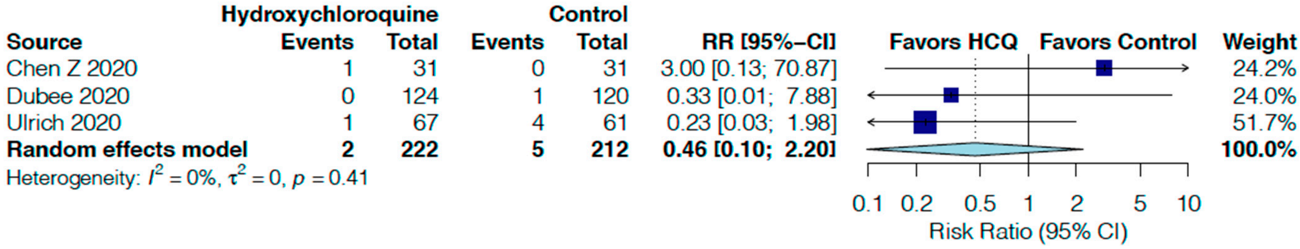

Supplementary Figure S14: Effect of hydroxychloroquine on headache in hospitalized COVID-19 patients

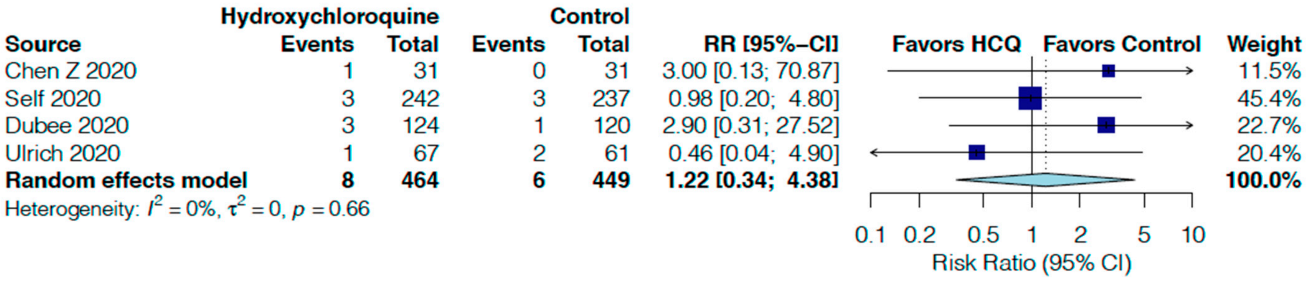

Supplementary Figure S15: Effect of hydroxychloroquine on QTc prolongation in hospitalized COVID-19 patients

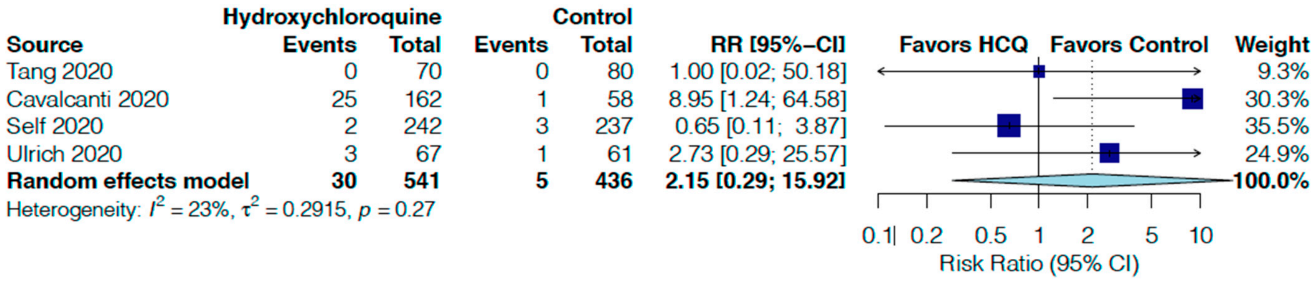

Supplementary Figure S16: Effect of hydroxychloroquine on anemia in hospitalized COVID-19 patients

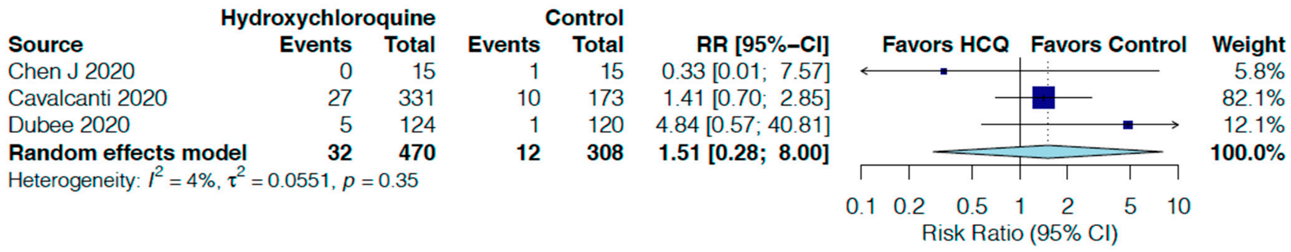

Supplementary Figure S17: Effect of hydroxychloroquine on ventricular tachycardia in hospitalized COVID-19 patients

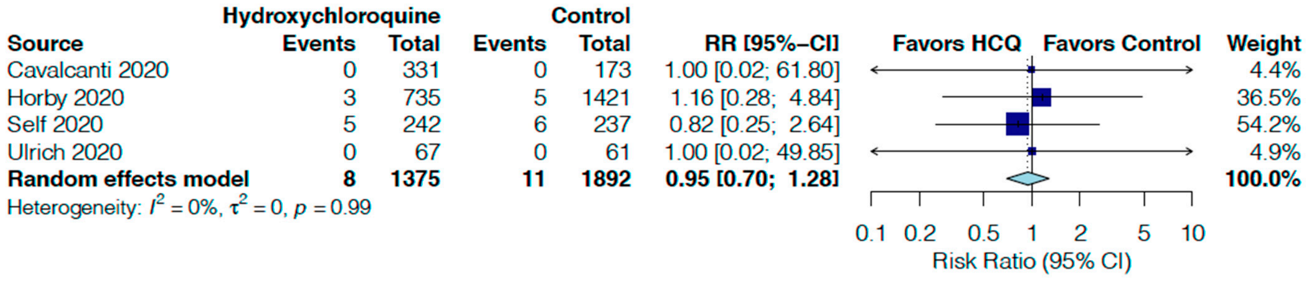

Supplementary Figure S18: Effect of hydroxychloroquine on leukopenia in hospitalized COVID-19 patients

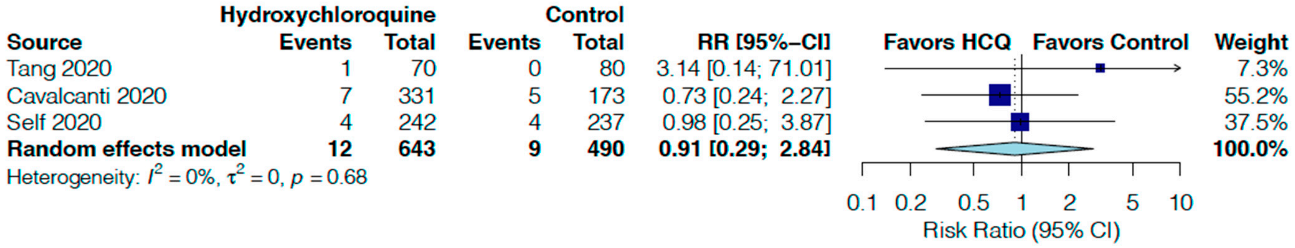

Supplementary Figure S19: Effect of hydroxychloroquine on lymphocytopenia in hospitalized COVID-19 patients

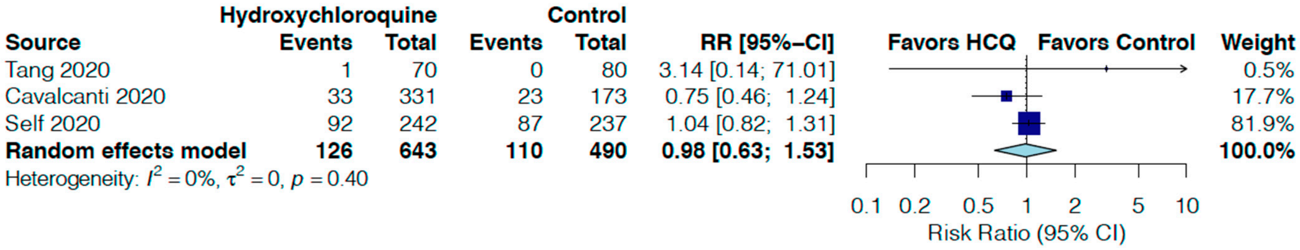

Supplementary Figure S20: Subgroup analyses

1. **All-cause mortality at 14 days:**

   a. **subgroups by control:**

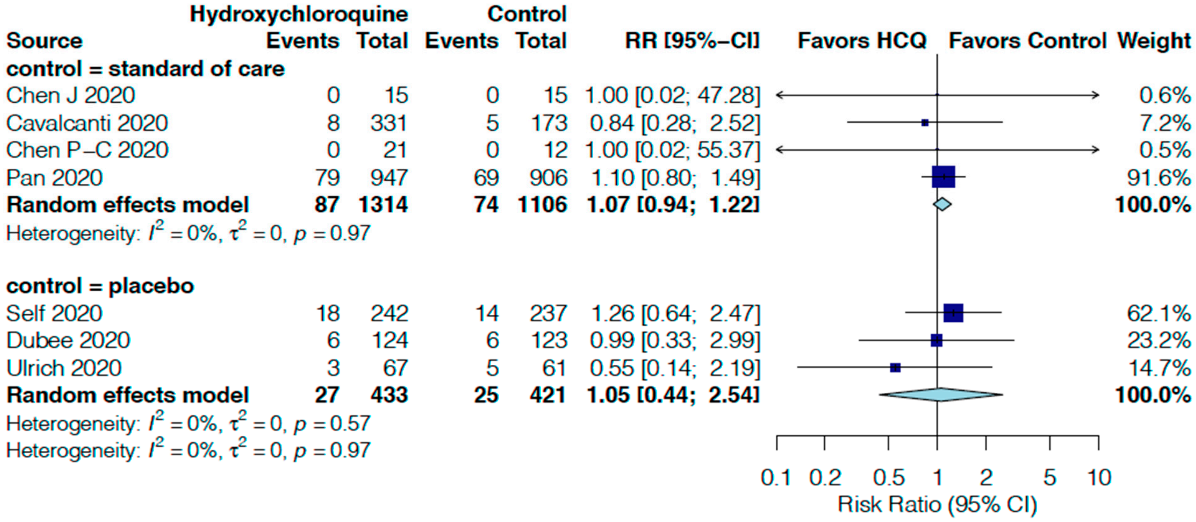

b. **subgroups by rob:**

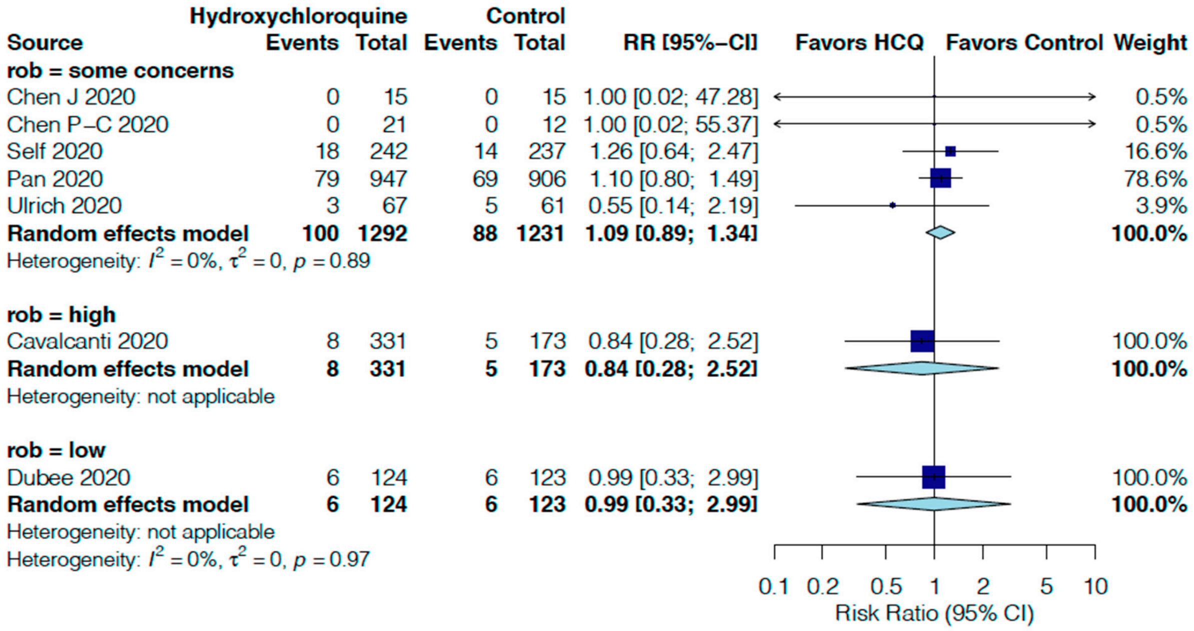

2. **All-cause mortality at 30 days:**

a. **subgroups by control:**

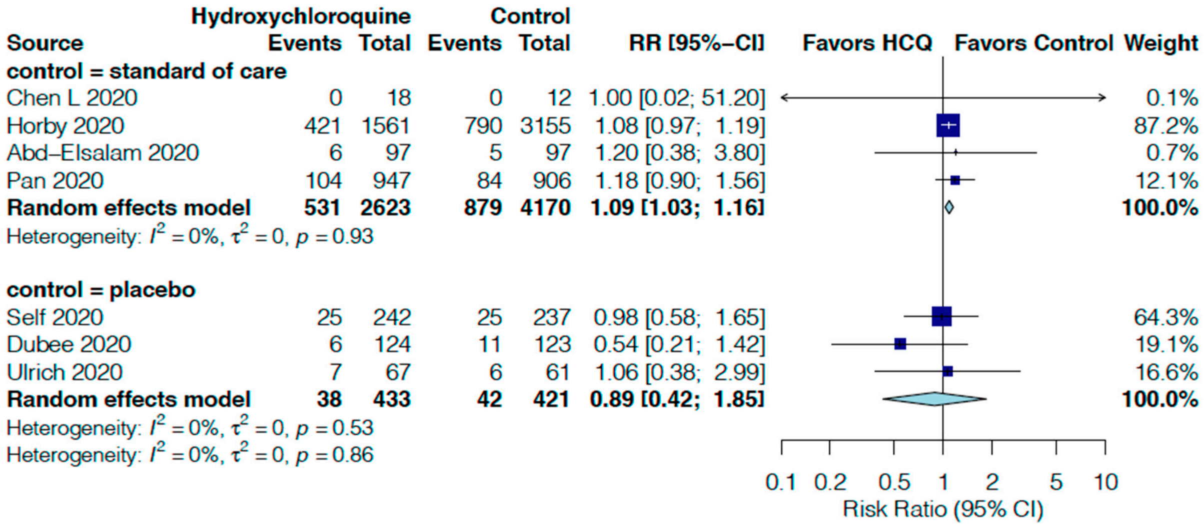

b. **subgroups by rob:**

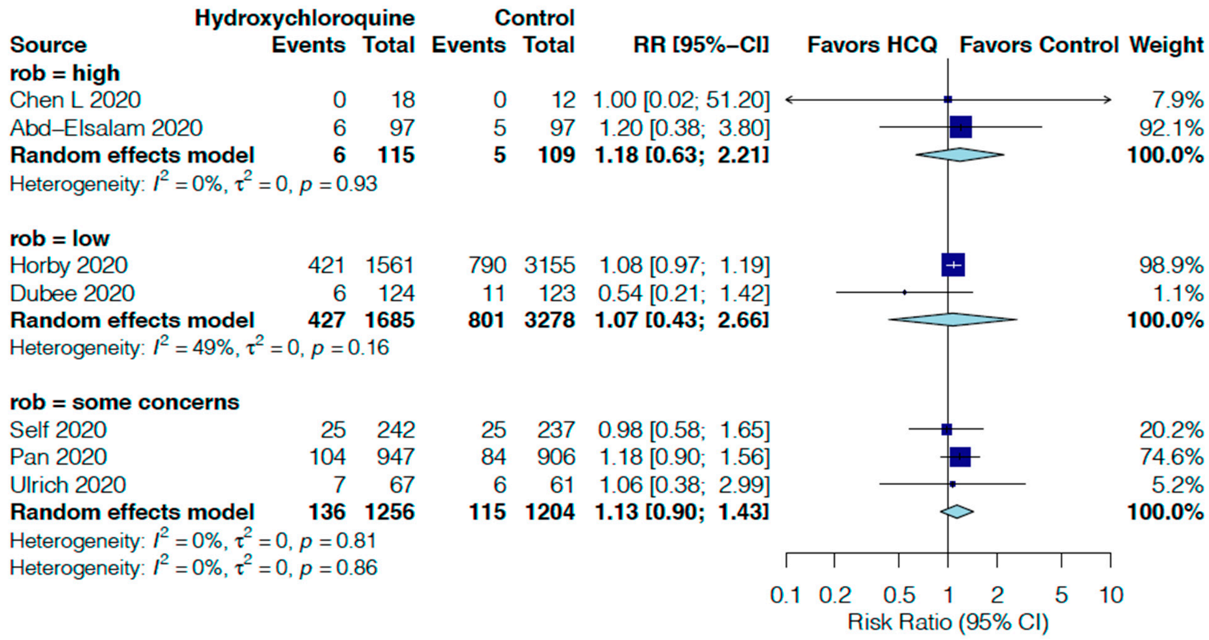

3. **Need for mechanical ventilation at 14 days:**

a. **subgroups by control:**

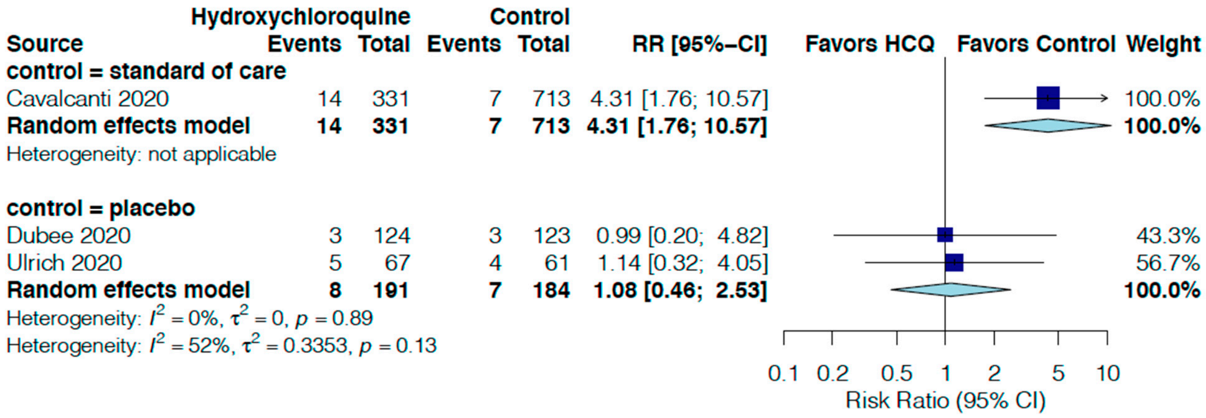

b. **subgroups by rob: NA**

4. **Need for mechanical ventilation at 30 days:**

a. **subgroups by control:**

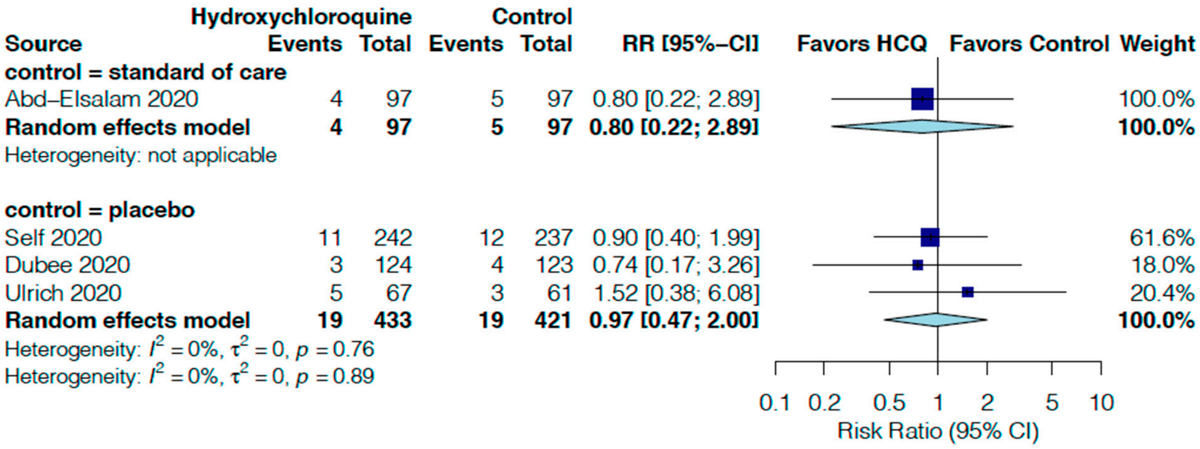

b. **subgroups by rob:**

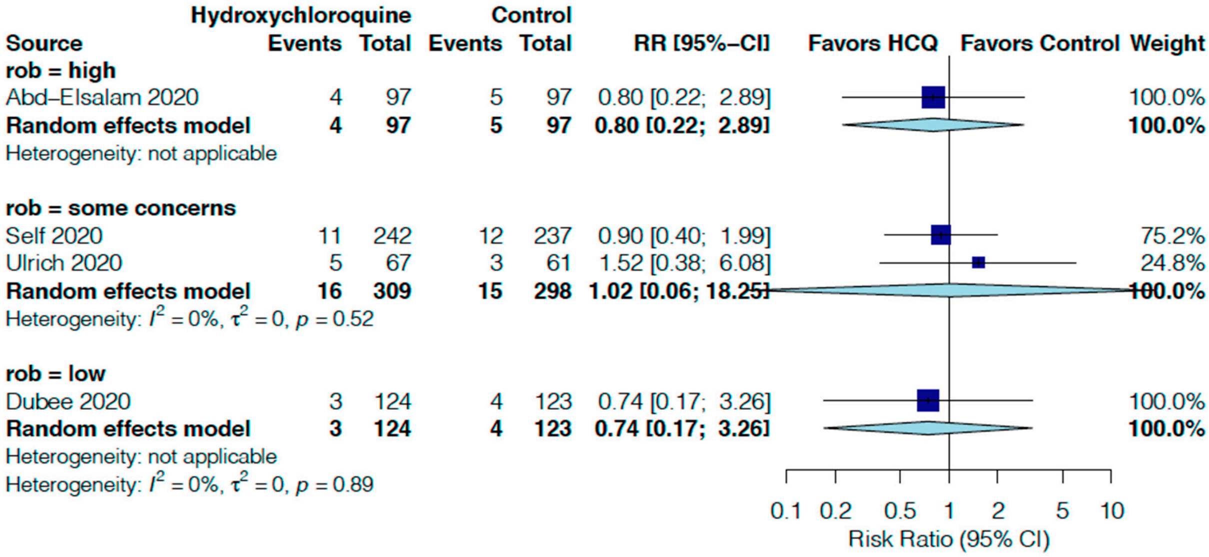

5. **High-flow nasal cannula or non-invasive ventilation at 14 days:**

a. **subgroups by control:**

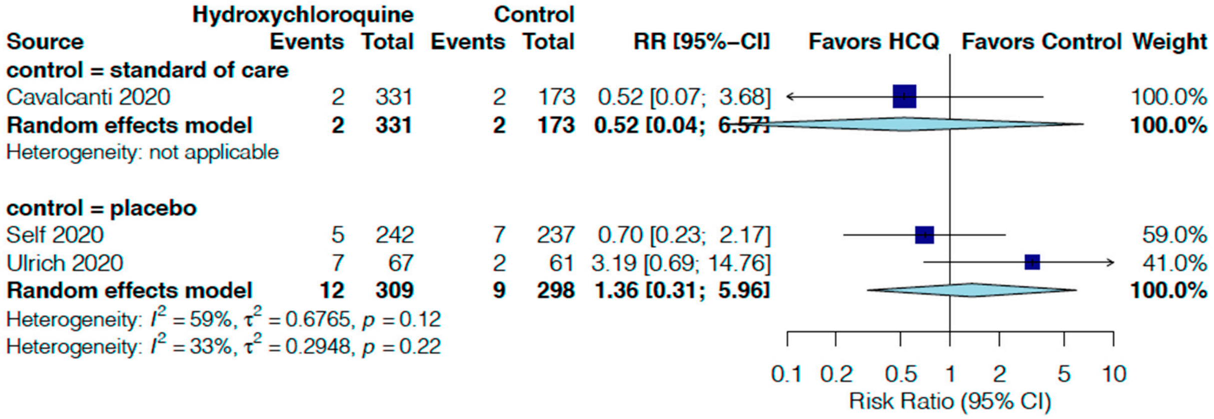

b. **subgroups by rob:**

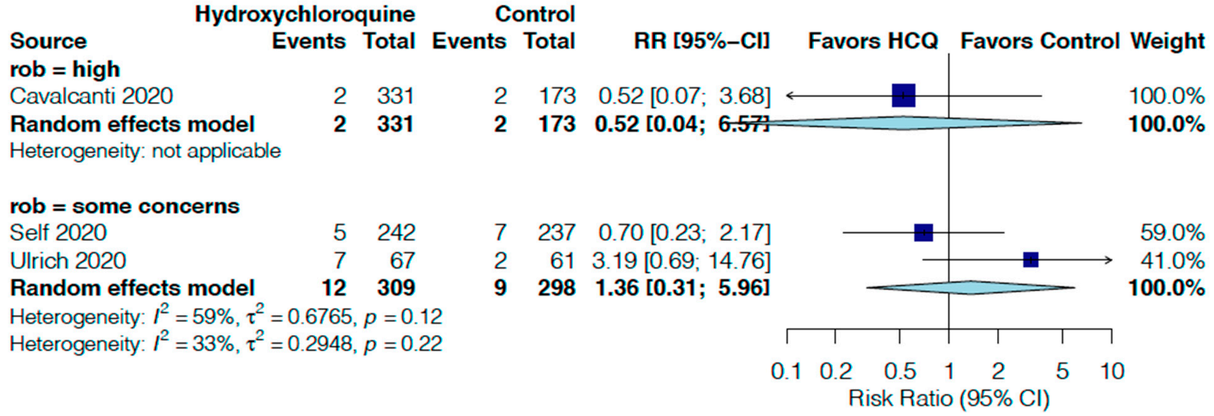

6. **Need of ICU: NA**

7. **Need for oxygen support at 14 days:**

a. **subgroups by control:**

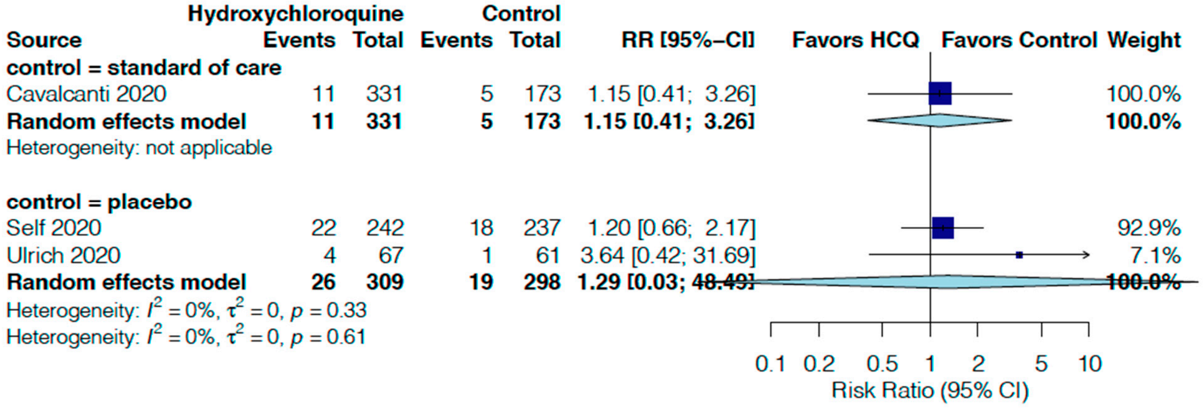

b. subgroups by rob:

Source	Hydroxychloroquine Events	Hydroxychloroquine Total	Control Events	Control Total	RR [95%–CI]	Weight
**rob = high**						
Cavalcanti 2020	11	331	5	173	1.15 [0.41; 3.26]	100.0%
**Random effects model**	**11**	**331**	**5**	**173**	**1.15 [0.41; 3.26]**	**100.0%**
Heterogeneity: not applicable						
**rob = some concerns**						
Self 2020	22	242	18	237	1.20 [0.66; 2.17]	92.9%
Ulrich 2020	4	67	1	61	3.64 [0.42; 31.69]	7.1%
**Random effects model**	**26**	**309**	**19**	**298**	**1.29 [0.03; 48.49]**	**100.0%**
Heterogeneity: $I^2 = 0\%$, $\tau^2 = 0$, $p = 0.33$						
Heterogeneity: $I^2 = 0\%$, $\tau^2 = 0$, $p = 0.61$						

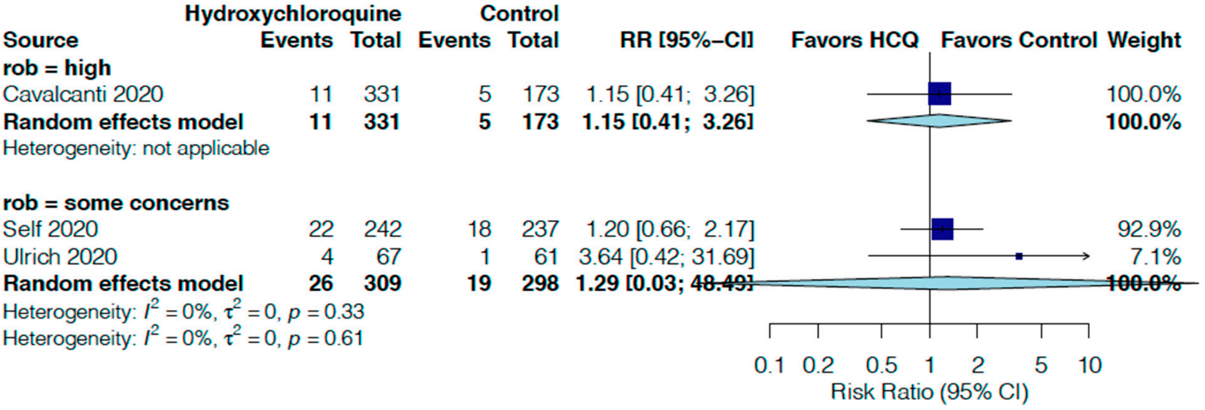

8. Clinical recovery: NA

9. Clinical worsening (death or invasive mechanical ventilation): NA

10. Discharge:

a. subgroups by control:

Source	Hydroxychloroquine Events	Hydroxychloroquine Total	Control Events	Control Total	RR [95%–CI]	Weight
**control = placebo**						
Self 2020	191	242	185	237	1.01 [0.92; 1.11]	49.5%
Dubee 2020	90	123	85	123	1.06 [0.90; 1.24]	31.4%
Ulrich 2020	41	67	47	61	0.79 [0.63; 1.00]	19.0%
**Random effects model**	**322**	**432**	**317**	**421**	**0.97 [0.69; 1.37]**	**100.0%**
Heterogeneity: $I^2 = 53\%$, $\tau^2 = 0.0126$, $p = 0.12$						
**control = standard of care**						
Horby 2020	931	1561	1983	3155	0.95 [0.90; 1.00]	100.0%
**Random effects model**	**931**	**1561**	**1983**	**3155**	**0.95 [0.90; 1.00]**	**100.0%**
Heterogeneity: not applicable						

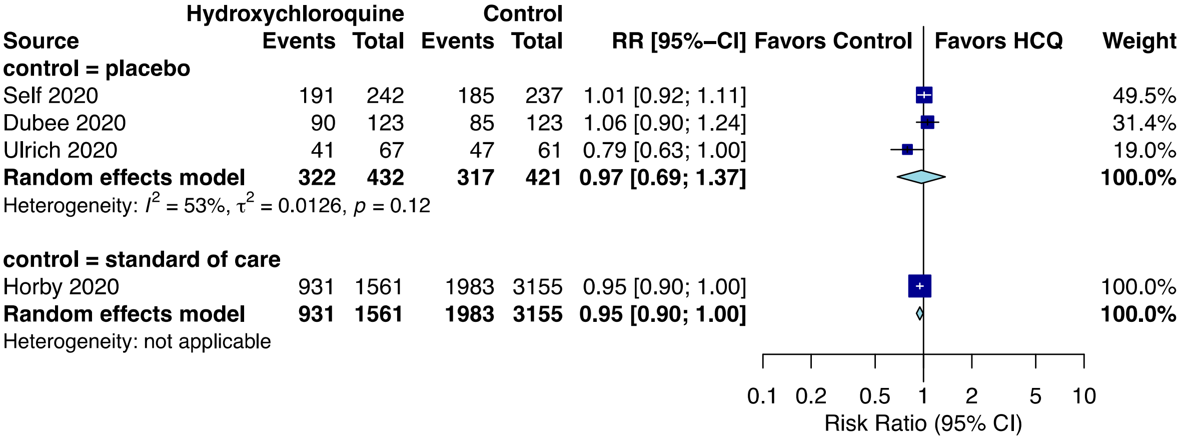

b. subgroups by rob:

Source	Hydroxychloroquine Events	Hydroxychloroquine Total	Control Events	Control Total	RR [95%–CI]	Weight
**rob = low**						
Horby 2020	931	1561	1983	3155	0.95 [0.90; 1.00]	67.0%
Dubee 2020	90	123	85	123	1.06 [0.90; 1.24]	33.0%
**Random effects model**	**1021**	**1684**	**2068**	**3278**	**0.98 [0.53; 1.78]**	**100.0%**
Heterogeneity: $I^2 = 40\%$, $\tau^2 = 0.0024$, $p = 0.20$						
**rob = some concerns**						
Self 2020	191	242	185	237	1.01 [0.92; 1.11]	72.3%
Ulrich 2020	41	67	47	61	0.79 [0.63; 1.00]	27.7%
**Random effects model**	**232**	**309**	**232**	**298**	**0.92 [0.20; 4.12]**	**100.0%**
Heterogeneity: $I^2 = 71\%$, $\tau^2 = 0.0208$, $p = 0.06$						

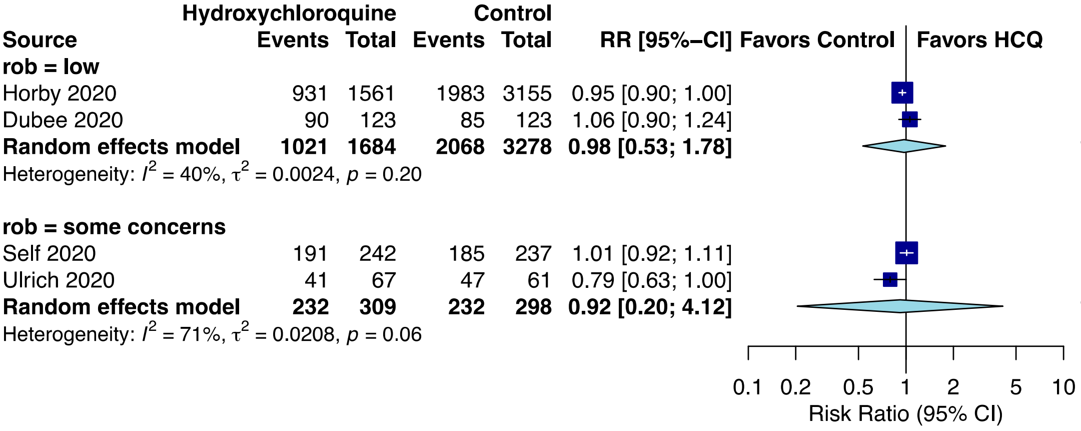

11. Radiological progression of pneumonia: NA

12. Virologic clearance at 14 days:

a. subgroups by control:

Source	Hydroxychloroquine Events	Hydroxychloroquine Total	Control Events	Control Total	RR [95%–CI]	Weight
**control = standard of care**						
Chen J 2020	15	15	15	15	1.00 [0.88; 1.14]	44.0%
Chen P–C 2020	17	21	9	12	1.08 [0.73; 1.59]	4.9%
Kamran 2020	244	349	110	151	0.96 [0.85; 1.08]	51.1%
**Random effects model**	**276**	**385**	**134**	**178**	**0.98 [0.90; 1.07]**	**100.0%**
Heterogeneity: $I^2 = 0\%$, $\tau^2 = 0$, $p = 0.80$						
**control = placebo**						
Ulrich 2020	8	67	10	61	0.73 [0.31; 1.73]	100.0%
**Random effects model**	**8**	**67**	**10**	**61**	**0.73 [0.31; 1.73]**	**100.0%**
Heterogeneity: not applicable						
Heterogeneity: $I^2 = 0\%$, $\tau^2 = 0$, $p = 0.82$						

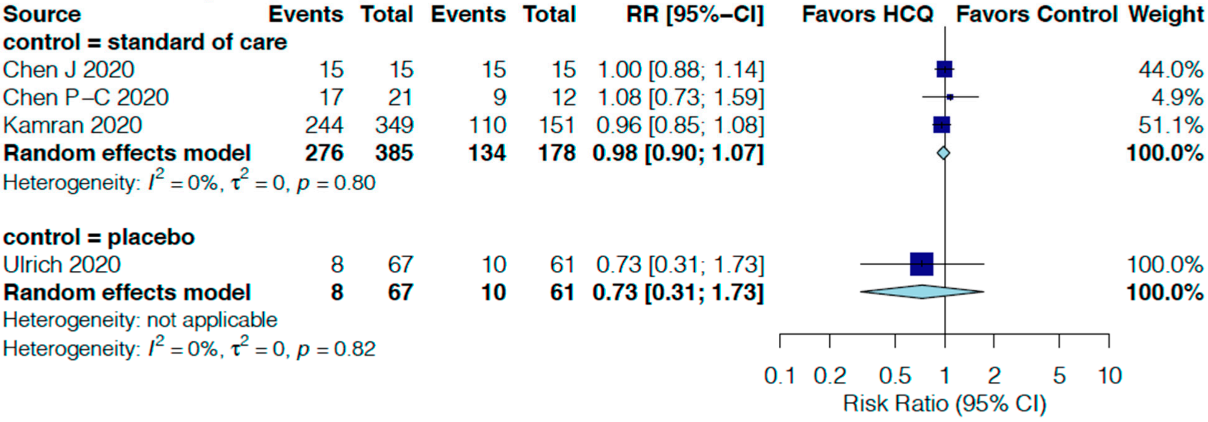

b. subgroups by rob: NA

13. Adverse events:

a. **subgroups by control:**

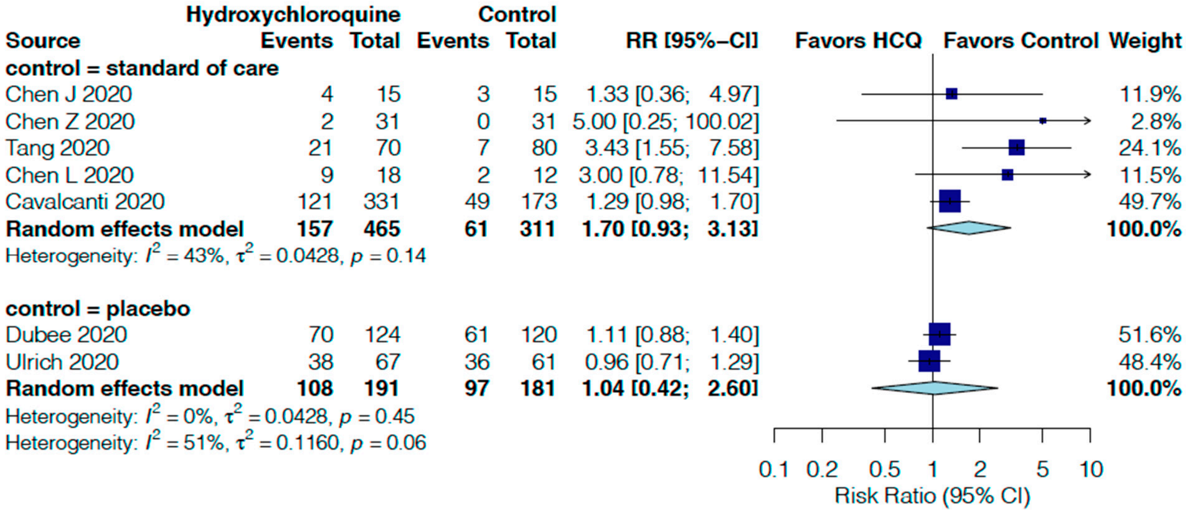

b. **subgroups by rob:**

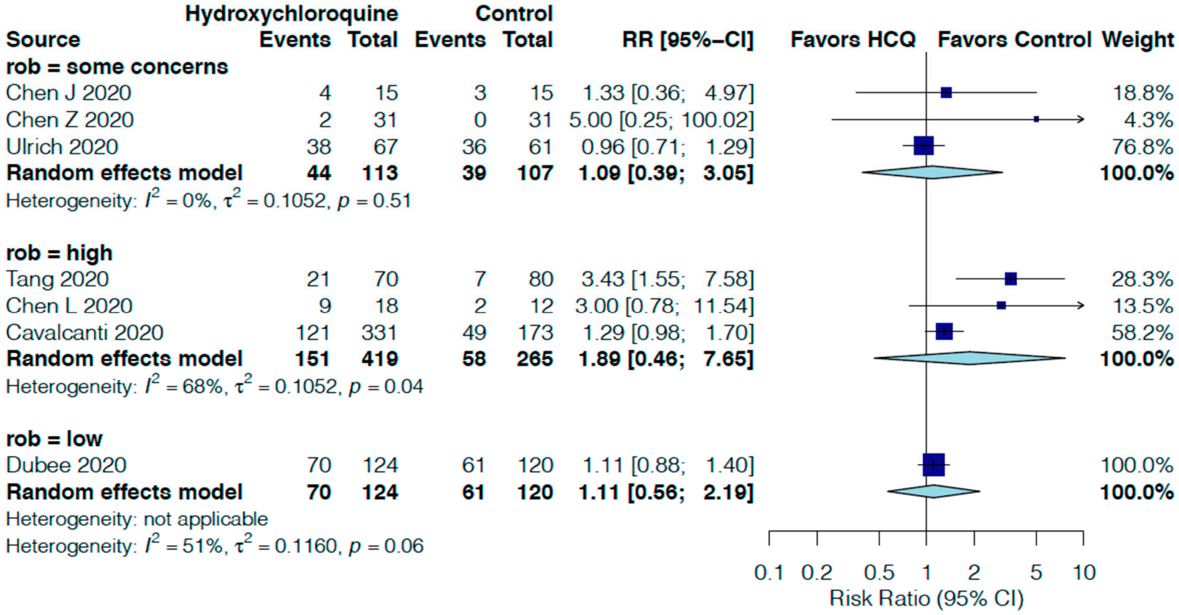

14. Severe adverse events:

a. **subgroups by control:**

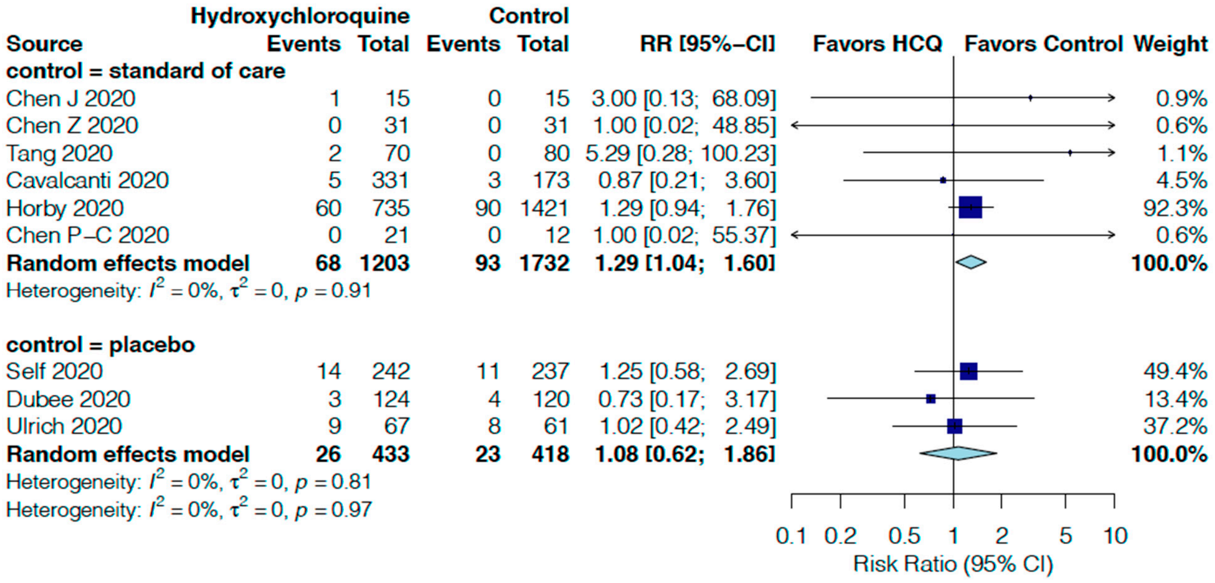

b. **subgroups by rob:**

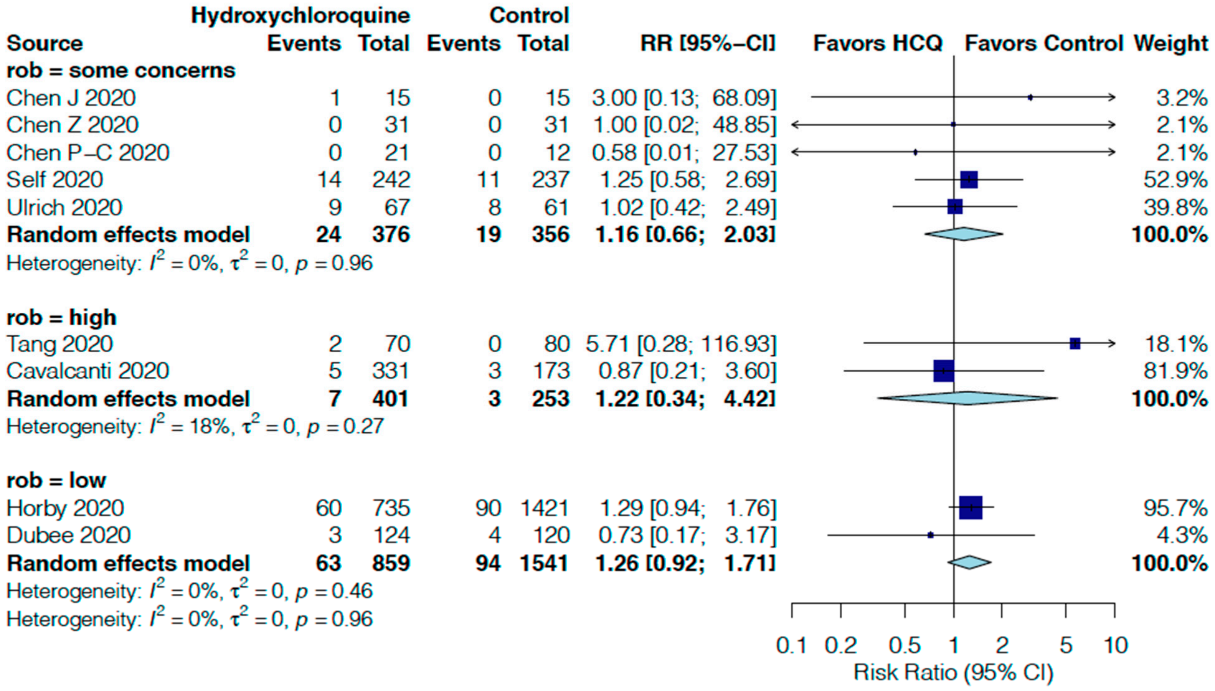

15. Gastrointestinal adverse events:

a. **subgroups by control:**

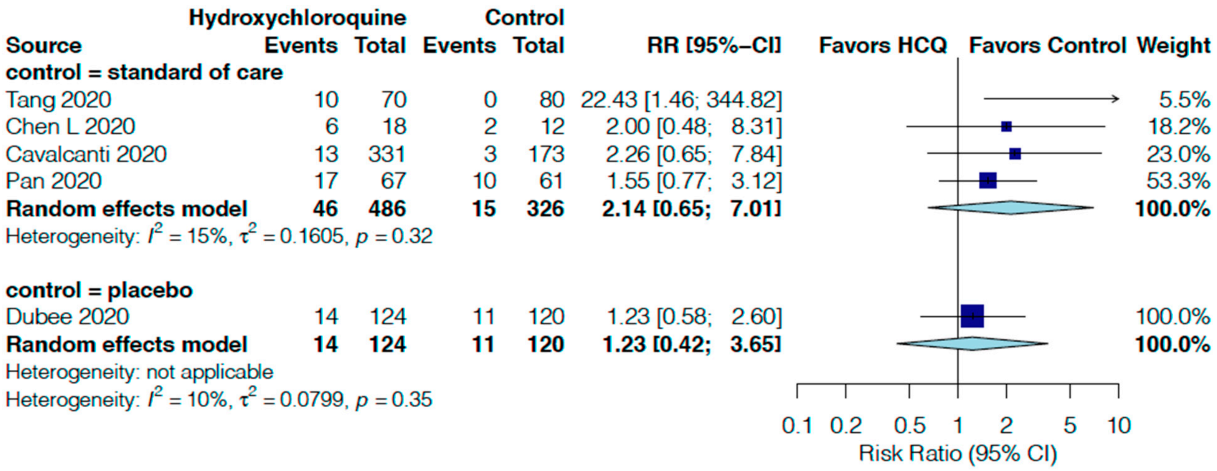

b. **subgroups by rob:**

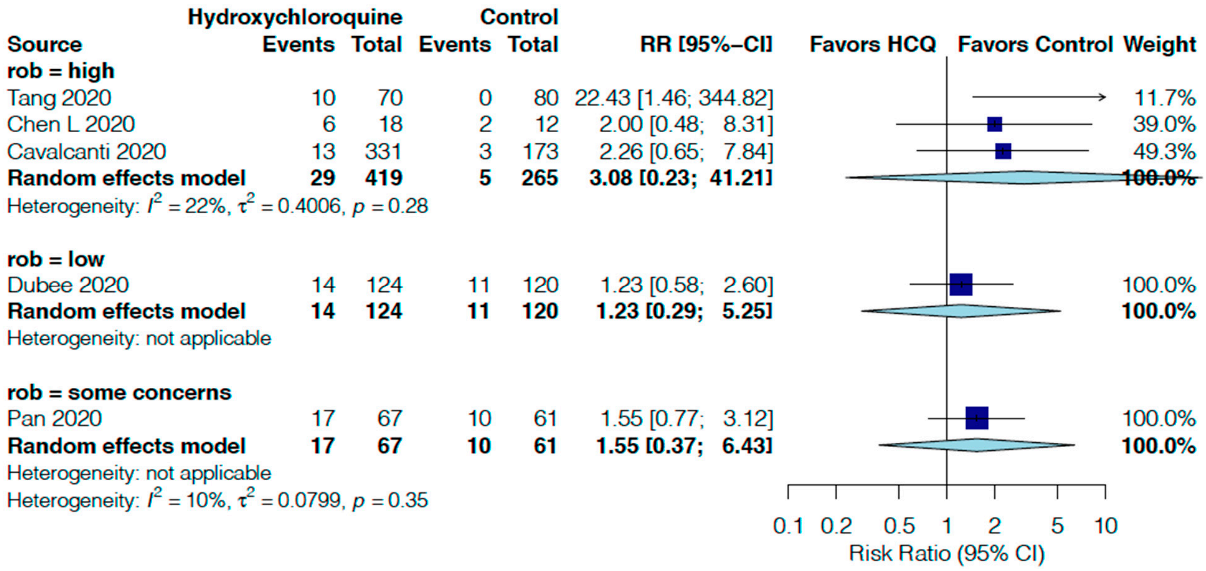

16. Abnormal liver function:

a. **subgroups by control:**

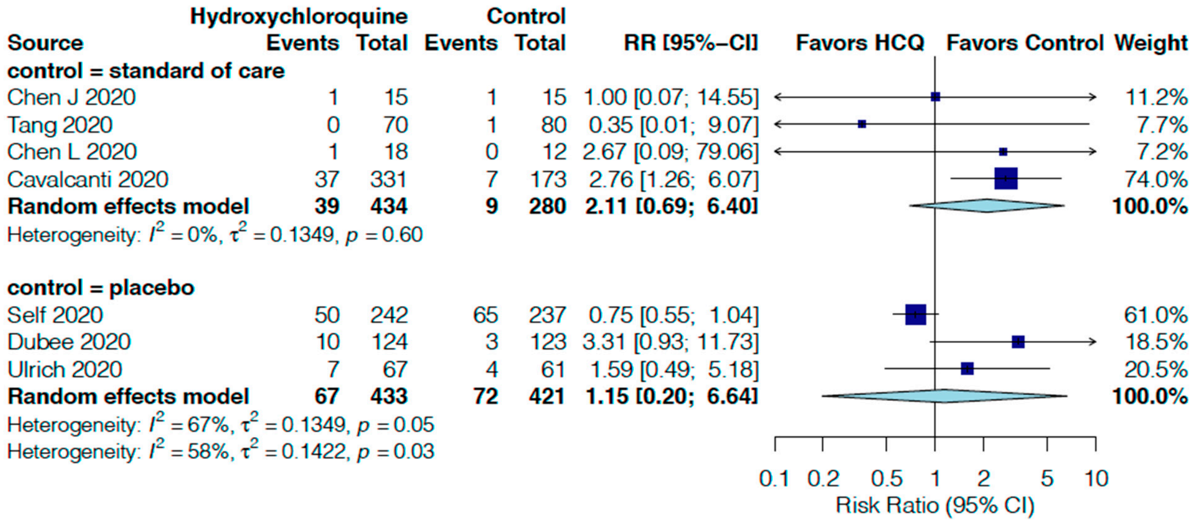

b. **subgroups by rob:**

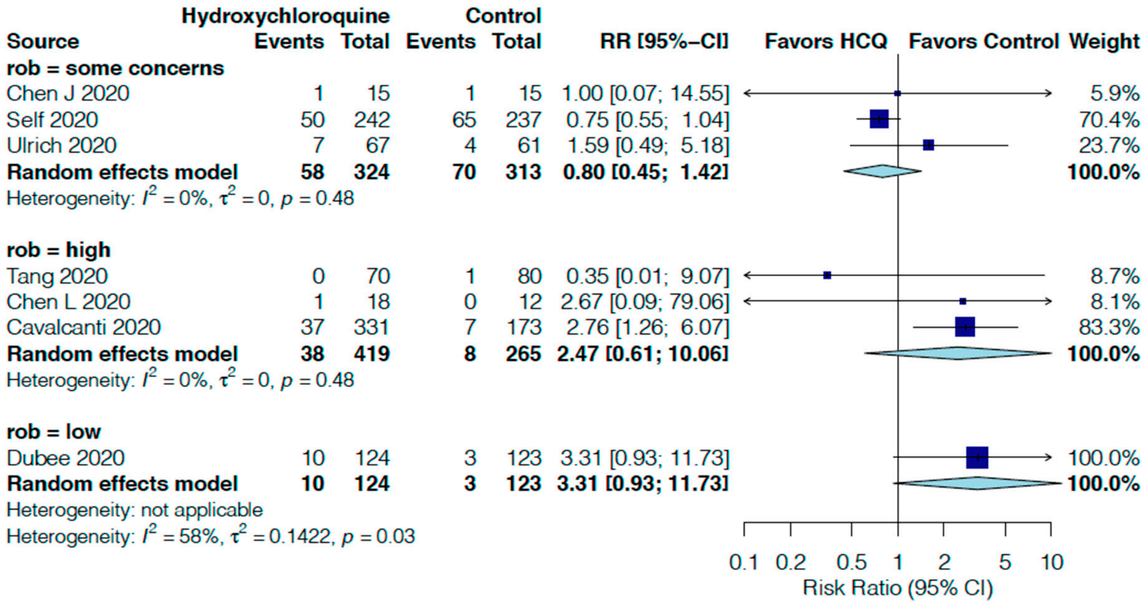

17. Rash:

a. subgroups by control:

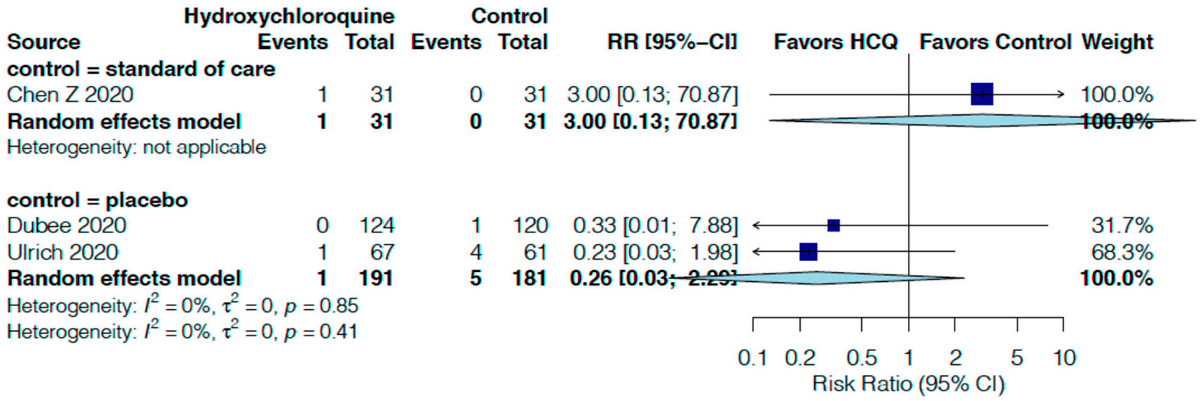

b. subgroups by rob:

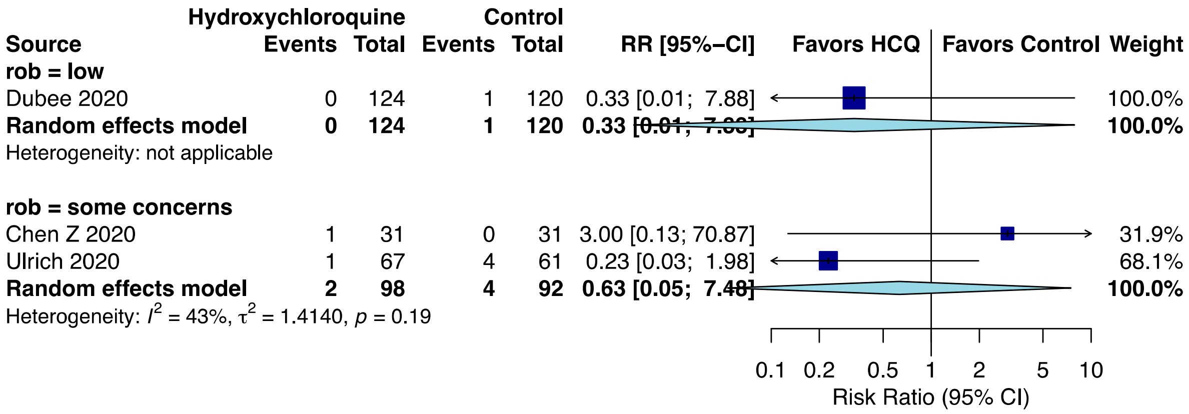

18. Headache:

a. subgroups by control:

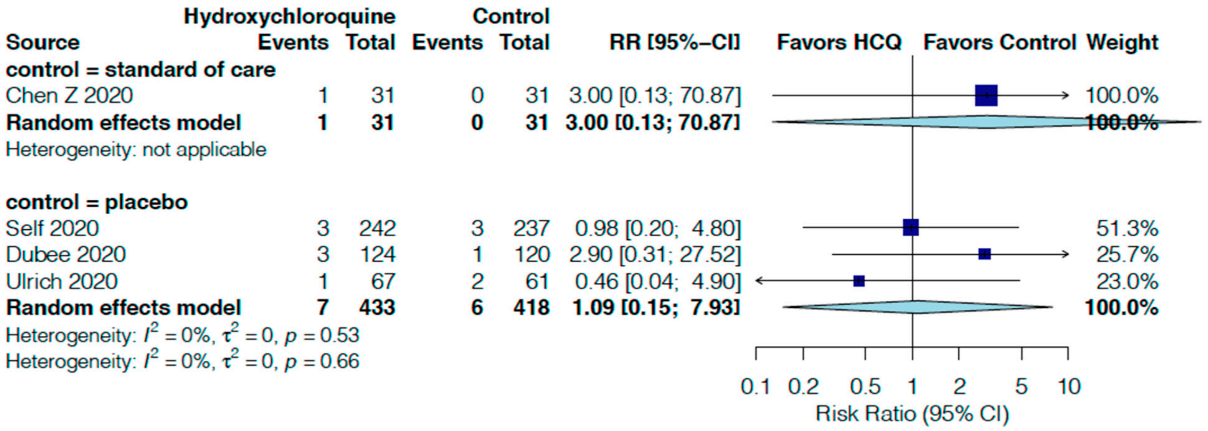

b. subgroups by rob:

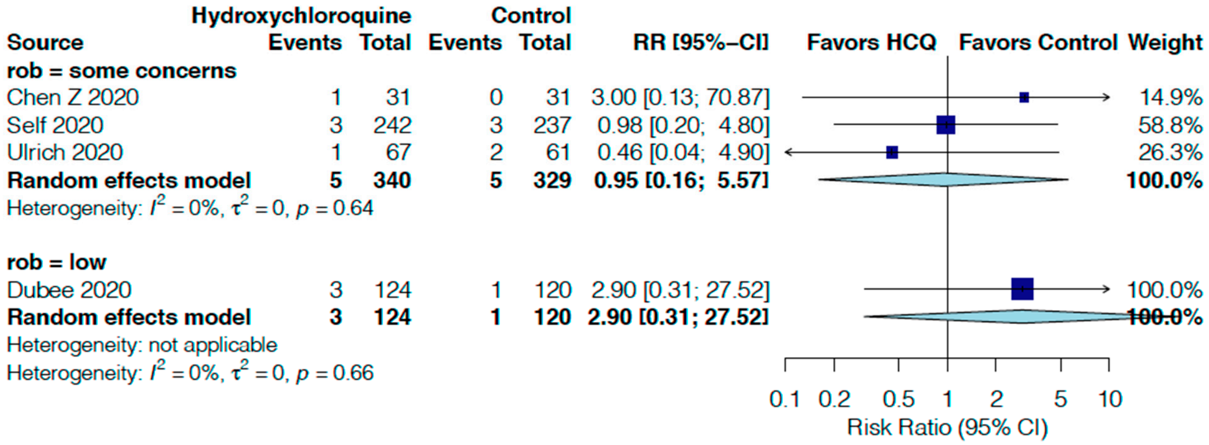

19. QTc prolongation:

a. subgroups by control:

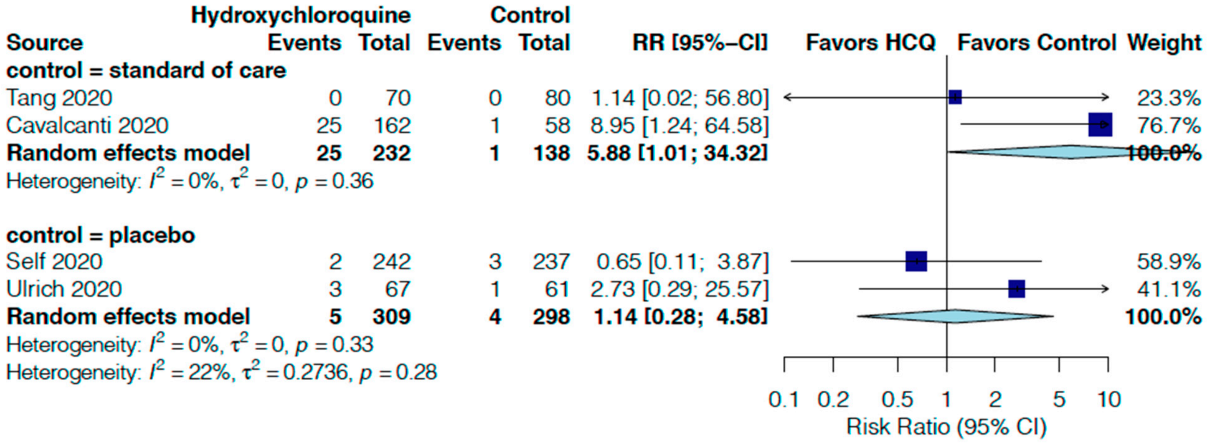

b. **subgroups by rob:**

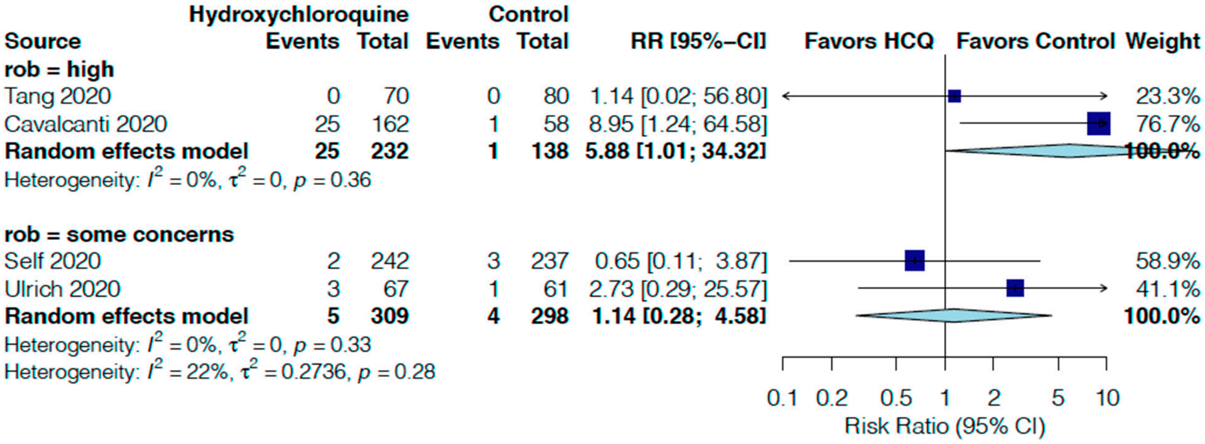

20. Anemia:

a. **subgroups by control:**

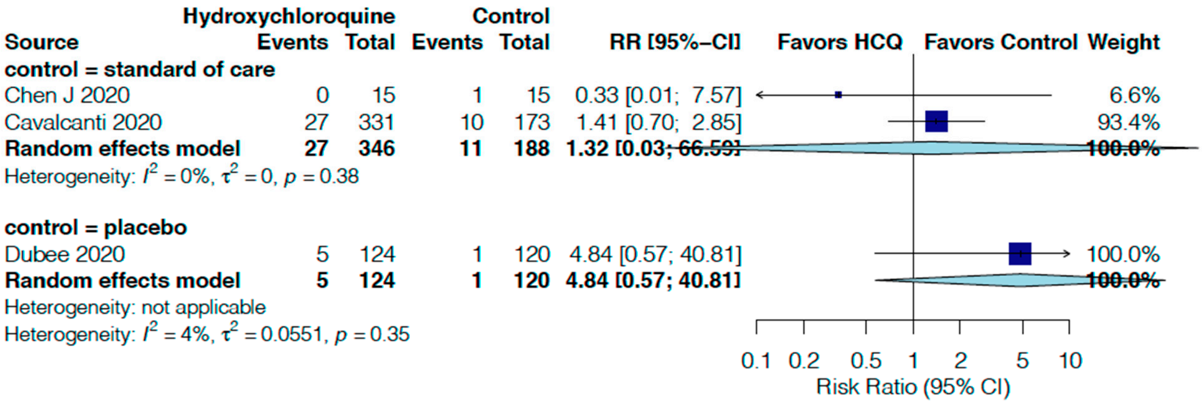

b. **subgroups by rob:**

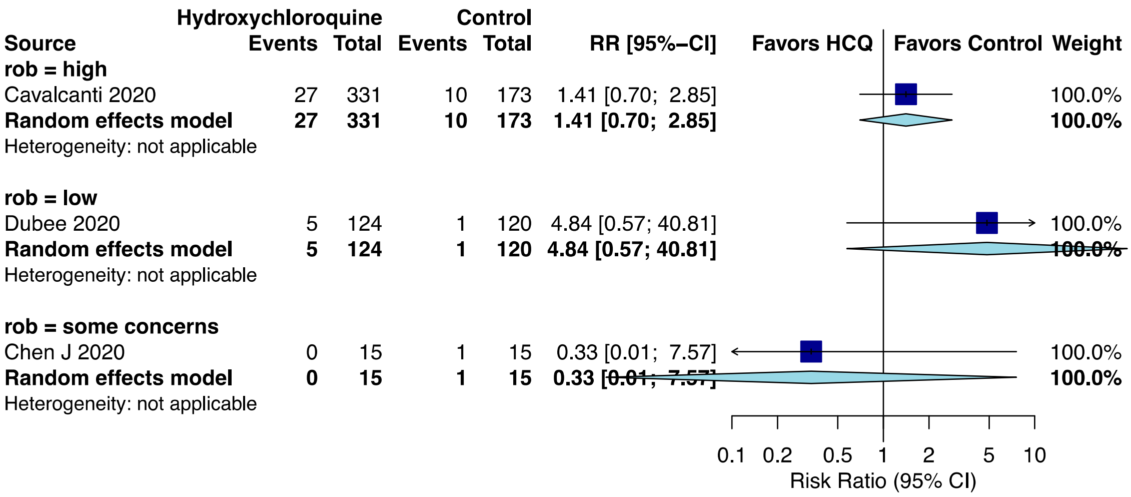

21. Ventricular tachycardia:

a. subgroups by control:

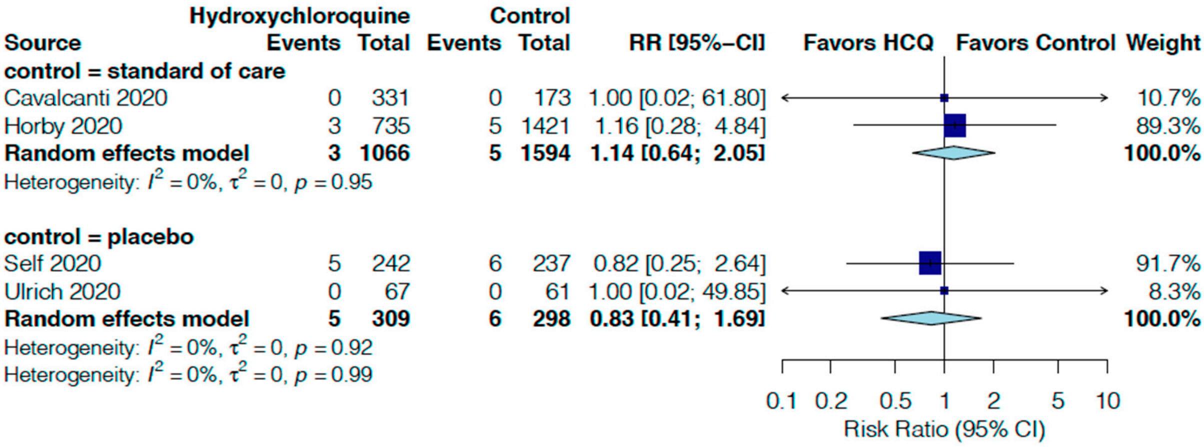

b. subgroups by rob:

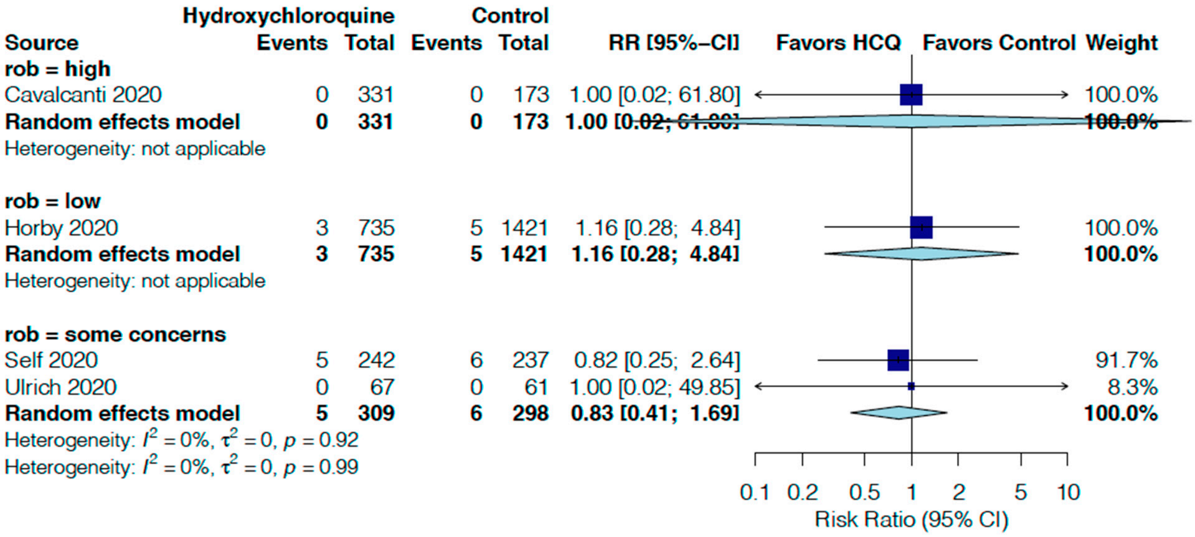

22. Leukopenia:

a. subgroups by control:

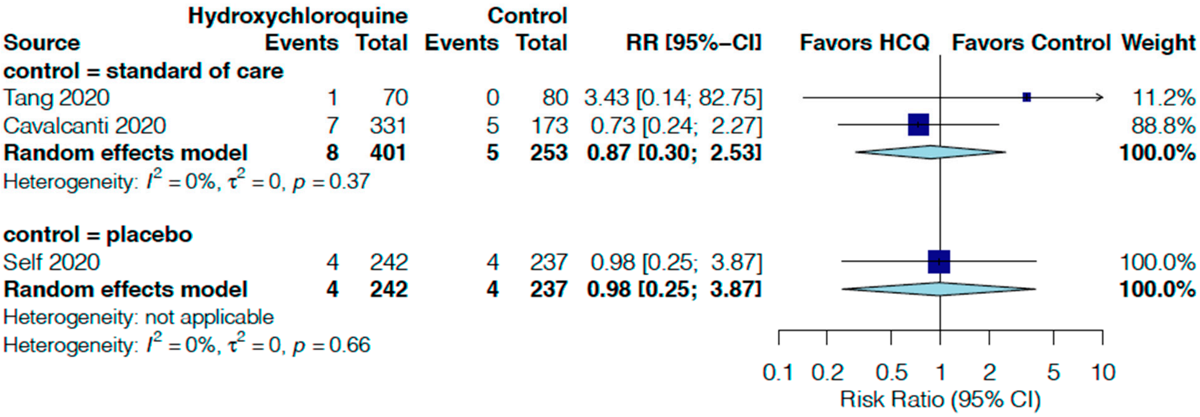

b. subgroups by rob:

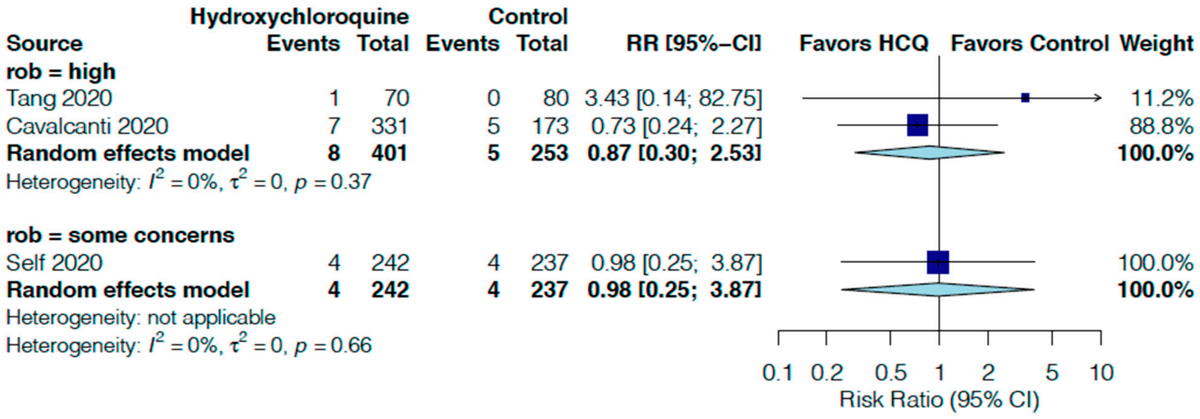

23. Lymphocytopenia:

a. subgroups by control:

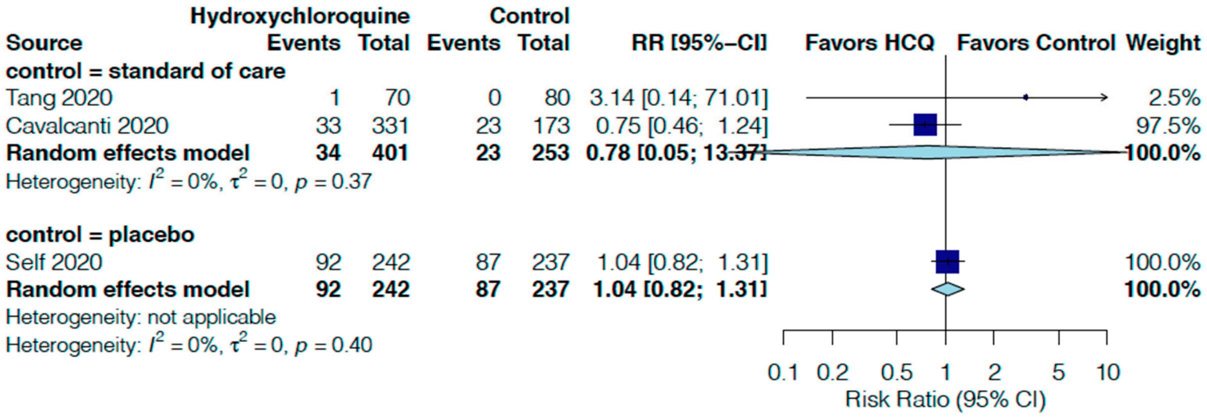

b. subgroups by rob:

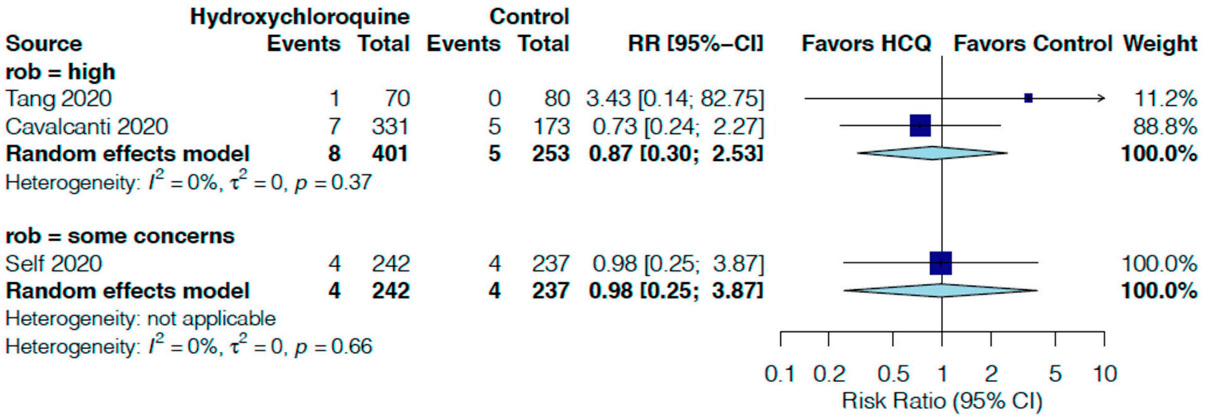

Section/topic	#	Checklist item	Reported on page #
**TITLE**			
Title	1	Identify the report as a systematic review, meta-analysis, or both.	1
**ABSTRACT**			
Structured summary	2	Provide a structured summary including, as applicable: background; objectives; data sources; study eligibility criteria, participants, and interventions; study appraisal and synthesis methods; results; limitations; conclusions and implications of key findings; systematic review registration number.	1
**INTRODUCTION**			
Rationale	3	Describe the rationale for the review in the context of what is already known.	1,2
Objectives	4	Provide an explicit statement of questions being addressed with reference to participants, interventions, comparisons, outcomes, and study design (PICOS).	2
**METHODS**			
Protocol and registration	5	Indicate if a review protocol exists, if and where it can be accessed (e.g., Web address), and, if available, provide registration information including registration number.	–
Eligibility criteria	6	Specify study characteristics (e.g., PICOS, length of follow-up) and report characteristics (e.g., years considered, language, publication status) used as criteria for eligibility, giving rationale.	2
Information sources	7	Describe all information sources (e.g., databases with dates of coverage, contact with study authors to identify additional studies) in the search and date last searched.	2
Search	8	Present full electronic search strategy for at least one database, including any limits used, such that it could be repeated.	2
Study selection	9	State the process for selecting studies (i.e., screening, eligibility, included in systematic review, and, if applicable, included in the meta-analysis).	2
Data collection process	10	Describe method of data extraction from reports (e.g., piloted forms, independently, in duplicate) and any processes for obtaining and confirming data from investigators.	2
Data items	11	List and define all variables for which data were sought (e.g., PICOS, funding sources) and any assumptions and simplifications made.	2
Risk of bias in individual studies	12	Describe methods used for assessing risk of bias of individual studies (including specification of whether this was done at the study or outcome level), and how this information is to be used in any data synthesis.	2
Summary measures	13	State the principal summary measures (e.g., risk ratio, difference in means).	2,3
Synthesis of results	14	Describe the methods of handling data and combining results of studies, if done, including measures of consistency (e.g., I^2) for each meta-analysis.	2,3

Section/topic	#	Checklist item	Reported on page #
Risk of bias across studies	15	Specify any assessment of risk of bias that may affect the cumulative evidence (e.g., publication bias, selective reporting within studies).	5,6
Additional analyses	16	Describe methods of additional analyses (e.g., sensitivity or subgroup analyses, meta-regression), if done, indicating which were pre-specified.	5,6
**RESULTS**			
Study selection	17	Give numbers of studies screened, assessed for eligibility, and included in the review, with reasons for exclusions at each stage, ideally with a flow diagram.	3; Figure 1
Study characteristics	18	For each study, present characteristics for which data were extracted (e.g., study size, PICOS, follow-up period) and provide the citations.	3; Table 1
Risk of bias within studies	19	Present data on risk of bias of each study and, if available, any outcome level assessment (see item 12).	9,10; Figure 2
Results of individual studies	20	For all outcomes considered (benefits or harms), present, for each study: (a) simple summary data for each intervention group (b) effect estimates and confidence intervals, ideally with a forest plot.	10,11; Figures 3-6; Figures S1-S19
Synthesis of results	21	Present results of each meta-analysis done, including confidence intervals and measures of consistency.	10,11
Risk of bias across studies	22	Present results of any assessment of risk of bias across studies (see Item 15).	9,10; Table 2
Additional analysis	23	Give results of additional analyses, if done (e.g., sensitivity or subgroup analyses, meta-regression [see Item 16]).	11
**DISCUSSION**			
Summary of evidence	24	Summarize the main findings including the strength of evidence for each main outcome; consider their relevance to key groups (e.g., healthcare providers, users, and policy makers).	14,15
Limitations	25	Discuss limitations at study and outcome level (e.g., risk of bias), and at review-level (e.g., incomplete retrieval of identified research, reporting bias).	15
Conclusions	26	Provide a general interpretation of the results in the context of other evidence, and implications for future research.	15
**FUNDING**			

Funding	27	Describe sources of funding for the systematic review and other support (e.g., supply of data); role of funders for the systematic review.	16
